# Barriers and facilitators for early and exclusive breastfeeding in health facilities in Sub-Saharan Africa: a systematic review

**DOI:** 10.1186/s41256-021-00206-2

**Published:** 2021-07-06

**Authors:** Mai-Lei Woo Kinshella, Sarina Prasad, Tamanda Hiwa, Marianne Vidler, Alinane Linda Nyondo-Mipando, Queen Dube, David Goldfarb, Kondwani Kawaza

**Affiliations:** 1 Department of Obstetrics and Gynaecology, BC Children’s and Women’s Hospital and University of British Columbia, Vancouver, Canada; 2 Department of Pediatrics and Child Health, College of Medicine, University of Malawi, Blantyre, Malawi; 3 School of Public Health and Family Medicine, Department of Health Systems and Policy, College of Medicine, University of Malawi, Blantyre, Malawi; 4 Queen Elizabeth Central Hospital, Pediatrics, Blantyre, Malawi; 5 Department of Pathology and Laboratory Medicine, BC Children’s and Women’s Hospital and University of British Columbia, Vancouver, Canada

**Keywords:** Breastfeeding, Breastfeeding support, Barriers, Facilitators, Health facilities, Africa south of the Sahara

## Abstract

**Background:**

Sub-Saharan Africa carries a disproportionate burden of under-five child deaths in the world and appropriate breastfeeding practices can support efforts to reduce child mortality rates. Health facilities are important in the promotion of early and exclusive breastfeeding. The purpose of this review was to examine facility-based barriers and facilitators to early and exclusive breastfeeding in Sub-Saharan Africa.

**Methods:**

A systematic search was conducted on Medline, Web of Science, CINAHL, African Journals Online and African Index Medicus from database inception to April 29, 2021 and primary research studies on breastfeeding practices in health facilities in Sub-Saharan Africa were included in the review. We assessed qualitative studies with the Critical Appraisal Skills Programme Qualitative Checklist and quantitative studies using the National Heart, Lung, and Blood Institute tool. The review protocol was registered to Prospero prior to conducting the review (CRD42020167414).

**Results:**

Of the 56 included studies, relatively few described health facility infrastructure and supplies-related issues (5, 11%) while caregiver factors were frequently described (35, 74%). Facility-based breastfeeding policies and guidelines were frequently available but challenged by implementation gaps, especially at lower health service levels. Facilitators included positive caregiver and health worker attitudes, knowledge and support during the postpartum period. Current studies have focused on caregiver factors, particularly around their knowledge and attitudes, while health facility infrastructure and supplies factors appear to be growing concerns, such as overcrowding and lack of privacy during breastfeeding counselling that lowers the openness and comfort of mothers especially those HIV-positive.

**Conclusion:**

There has been a dramatic rise in rates of facility births in Sub-Saharan Africa, which must be taken into account when considering the capacities of health facilities to support breastfeeding practices. As the number of facility births rise in Sub-Saharan Africa, so does the responsibility of skilled healthcare workers to provide the necessary breastfeeding support and advice to caregivers. Our review highlighted that health facility infrastructure, supplies and staffing appears to be a neglected area in breastfeeding promotion and a need to strengthen respectful maternity care in the delivery of breastfeeding counselling, particularly in supporting HIV-positive mothers within the context of Sub-Saharan Africa.

**Supplementary Information:**

The online version contains supplementary material available at 10.1186/s41256-021-00206-2.

## Introduction

Sub-Saharan Africa (SSA) carries a disproportionate burden of infant and child deaths, with 55–75% of under-five deaths in SSA attributed to inappropriate breastfeeding practices [1]. With a 35% prevalence of exclusive breastfeeding, rates in SSA are lower in comparison to other low- and middle-income countries (LMICs) (39%) [1] and only 18 out of 49 African countries are on track to meet the World Health Organization (WHO) Global Nutrition Targets 2025 to increase the rate to 50% [2]. Exclusive breastfeeding is well-recognized as one of the most effective interventions to improve newborn survival rates with life-long impacts on children beyond their infancy into adulthood [3–5] and is strongly recommended by the WHO [6].

A systematic review on barriers in LMICs found that inadequate antenatal care, poor maternal care during childbirth and return to livelihood activities were key challenges to exclusive breastfeeding [7]. The review also found that women who delivered at a health facility were more likely to engage in exclusive breastfeeding practices [7], which has also been found in studies across SSA [8–15]. These findings highlight the importance of health facilities in the promotion of appropriate breastfeeding practices, which is enshrined in the Baby-Friendly Hospital Initiative (BFHI). Launched in 1991 by the WHO and UNICEF and revised in 2018, BFHI outlines supportive policies and practices health facilities can undertake to protect and promote successful breastfeeding [16, 17]. While previous reviews broadly examined barriers and facilitators to exclusive or early initiation to breastfeeding [7, 18–23], they have not focused on factors modifiable at the health facility level, which is surprising due to the widespread promotion of BFHI. Encouragement to breastfeed at BFHI hospitals is associated with increased exclusive breastfeeding rates and overall longer breastfeeding duration [24, 25], leading some to suggest that variation in exclusive breastfeeding levels across Africa may be explained in part by varied success in implementing BFHI [2, 25].

With growing evidence of the beneficial effect of facility delivery on early and exclusive breastfeeding rates, particularly in BFHI hospitals, there is a need to deconstruct the facilitating factors and outstanding gaps at health facilities to strengthen sustainable and equitable breastfeeding support practices. Healthcare providers have key roles to strengthen breastfeeding in health systems as they influence and support decisions to breastfeed [26]. Thus, improving early and exclusive breastfeeding in SSA requires a deeper look into the role of skilled health care providers at facilities and their perceptions, knowledge and skills around breastfeeding support as well as an exploration of other facility-based barriers and facilitators. The aim of this systematic review is to determine what facility-based barriers and facilitators of early and exclusive breastfeeding support are present for newborns in SSA.

## Methods

This review has been developed in accordance with the Preferred Reporting Items for Systematic Reviews and Meta-Analysis (PRISMA) checklist (Table S1) [27]. A review protocol detailing the research question, search strategy, inclusion and exclusion criteria, quality assessment and strategy for data synthesis was developed in consultation with pediatric clinicians from Malawi (TW, QB, KK) to refine the scope of the review and ensure relevance to Sub-Saharan African contexts. The protocol was registered to PROSPERO (CRD42020167414) prior to conducting the review.

### Study inclusion and exclusion

Studies conducted with family members, health workers and institutions engaged with facility-based early or exclusive breastfeeding support services in SSA were included in the review (Table 1). We included intervention (controlled trials) and observational studies (cohort, case-controlled, cross-sectional, qualitative) reporting barriers and facilitators. We defined facility-based barriers and facilitators to be factors modifiable at the facility level that hindered or supported appropriate breastfeeding practices. Studies that did not involve services at a health facility in SSA, did not mention early initiation of breastfeeding or exclusive breastfeeding, focused on community-based breastfeeding programs or were conducted among African women and infants living in other regions were excluded. Due to limited capacity of the review team to comprehensively search non-English databases, non-English publications were excluded. Studies without primary data collection in a health facility were also excluded.

**Table 1:** Review framework

Population	Health workers supporting facilitation of early and/or exclusive breastfeeding, mothers with infants aged 0–6 months or asked to reflect about their experiences with exclusive breastfeeding during that time at a health facility, health facilities implementing early and/or exclusive breastfeeding strengthening programs
Intervention	Early and exclusive breastfeeding
Context	Health facilities in Sub-Saharan Africa
Comparisons	No breastfeeding promotion, N/A
Outcome	Facility-based barriers and facilitators to early and/or exclusive breastfeeding practices
Study design	Experimental studies (controlled trials) and observational studies (cohort, case-controlled, cross-sectional, qualitative)

### Search strategy

Searches were conducted on MEDLINE Ovid, Web of Science, Cumulative Index to Nursing and Allied Health, African Journals Online and African Index Medicus from database inception to April 29, 2021, with no limits applied. Searches were supplemented by scanning reference lists of papers included for review. Search terms broadly included breastfeeding, breastmilk, Sub-Saharan African countries, hospital, clinic, health facility, barrier, facilitator, factor and implementation (Table S2).

### Study selection

Titles and abstracts were independently screened by two reviewers (MWK, SP) according to the inclusion and exclusion criteria and selection disagreements were resolved by discussion. A third reviewer (TH) was asked to adjudicate in the absence of consensus. Full texts were then independently reviewed by the two reviewers (MWK, SP) with the third reviewer (TH) providing an independent assessment in any disagreements regarding eligibility.

### Data extraction

Details about the study design, country, health facility level, sample, method, breastfeeding practice, exclusive breastfeeding rate and early initiation rate where reported, barriers and facilitators were independently extracted by two reviewers into a data extraction sheet on Excel (Microsoft, Redmond, United States).

### Data analysis

The data extraction sheet was imported into Nvivo 12 (QSR International, Melbourne, Australia) where thematic analysis of barriers and facilitators according to health facilities infrastructure and supplies, supportive policies and policy implementation, health worker engagement and caregiver engagement was conducted. Results were reported as a narrative synthesis.

### Quality assessment

To access internal validity and overall study quality, we evaluated quantitative studies using the study quality assessment tools of the National Heart, Lung, and Blood Institute of the National Institutes of Health (NIH) [28] and qualitative studies using the CASP checklist [29]. An overall study rating based on critical concerns of internal validity was added to the CASP checklist to be similar to the NIH quality assessment tools. Studies were not excluded as a result of the quality assessment but noted in consideration of the results.

## Results

**Fig. 1:**
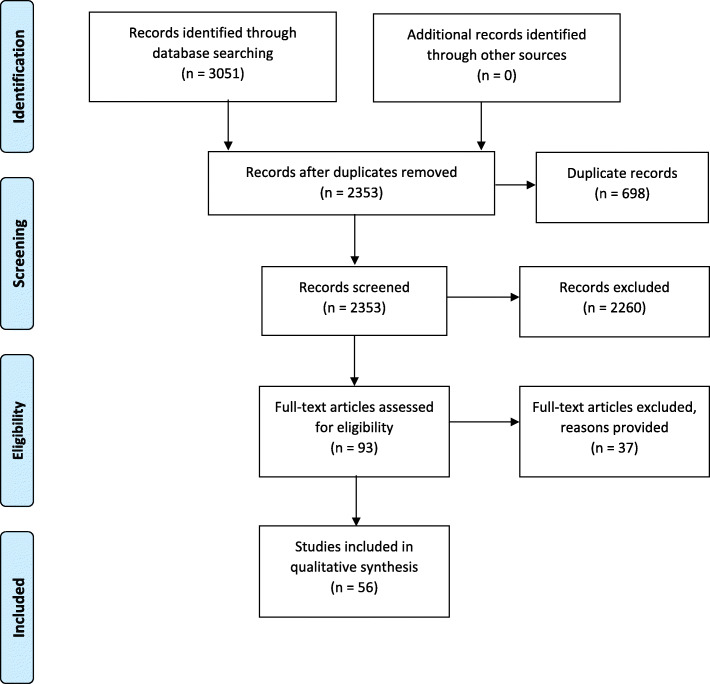
PRISMA flow diagram

We identified a total of 3051 records from our database searches (1233 from Medline, 1123 from Web of Science, 373 from CINAHL, 256 from African Index Medicus and 66 from African Journals Online). After removal of duplicates and screening against the eligibility criteria, 56 studies were included in the review (Fig. 1). Thirty-seven full-text articles were excluded for the following reasons: Research was not conducted in SSA (1 study), poor clarity regarding exclusive or complementary breastfeeding (3 studies), inability to isolate breastfeeding within a package of interventions (2 study), did not explore facility-based barriers and facilitators to breastfeeding (27 studies), duplicate (2 studies), outcomes reported in another publication (1 study) and was a conference proceeding (1 study) (Table S3).

Publication years of included studies ranged from 1995 to 2021, with the most studies conducted in 2019 (11 studies). There were 17 countries represented in this review: Democratic Republic of the Congo, Ethiopia, the Gambia, Ghana, Kenya, Lesotho, Malawi, Namibia, Niger, Nigeria, Rwanda, Somalia, South Africa, South Sudan, Tanzania, Uganda, and Zimbabwe. Nigeria was the most studied country with 12 studies included, followed by South Africa with 11 studies included. The studies varied from cross-sectional surveys and assessments (37 studies), qualitative study designs (15 studies), randomized controlled trials (2 studies), case-control studies (1 study) and pre-post studies with no control (1 study). Exclusive breastfeeding alone was assessed in 25 studies, early initiation breastfeeding alone in 13 studies and both practices were assessed in 18 studies. Finally, secondary-level district referral hospitals (16 studies) were the most targeted health level but primary-level (13 studies) and tertiary-level (11 studies) were also studied. A number of studies included a mix of the health facilities levels (14 studies) and health facility level was not reported in two studies. Characteristics of included studies are included in Table S4.

Four studies (7%) were assessed as good quality, 34 (61%) as fair and 18 (32%) as poor-quality studies. A majority of qualitative studies were rated fair to high quality (13 of 15, 87%) with clear descriptions of research objectives, appropriate research designs to understand perceptions or experiences, appropriate analysis and consideration of ethical issues. A majority of observational cohort or cross-sectional surveys were also rated as fair quality (21 of 37, 57%), though a substantial portion were rated poor quality (16 of 37, 43%) as they relied on self-reported breastfeeding intention or practices without follow-up, lack of clarity on questionnaires used and sampling methodology, and/or poor reporting of results. Both of the controlled intervention studies were rated good quality while the case-controlled and pre-post studies were both rated fair quality as adjustment for potential confounders were not conducted. Quality assessments by study are included in Table S5.

### Health facilities infrastructure and supplies

Of the 56 studies included in this review, 8 (14.3%) reported infrastructural barriers [30–37] while only one study (1.8%) reported facilitators [38]. Most frequently described barriers to postnatal breastfeeding support were overcrowding and lack of space [30–32, 34–36]. For example, a study from Ghana reported that increases in caesarean deliveries was associated with overcrowding and insufficient equipment, which led to moving new mothers out of delivery quickly to make room for the next woman in labour [30]. A study from Somalia also reported a lack of space in the maternity ward, which was associated with shorter stays and less time for counselling [32]. Lack of privacy, a quiet place to breastfeed, availability of chairs for mothers to sit and breastfeed in and unreliable access to water and electrical power were also infrastructural challenges to breastfeeding at health facilities [33, 35–37]. For example, a study from Zimbabwe highlighted how health workers asked mothers questions related to their HIV status while other patients were listening, which compromised confidentiality and decreased the likelihood of initiating breastfeeding [36]. In the one study that described infrastructural facilitators, the researchers in Tanzania reported that wall clocks and cell phone alarms supported regular timing feedings of low birthweight infants [38].

### Supportive policies and policy implementation

Almost a third of studies (18 of 56, 32.1%) reported policy-related barriers [30–32, 34, 35, 38–50] while 20 studies (35.7%) described facilitators [30, 31, 33–35, 37, 39–43, 47, 50–56]. Poor leadership and management structures were described as barriers to effective facility-based breastfeeding [30, 40, 47, 50], such as when hospital management felt that BFHI was extra work to implement. Also frequently reported was limited implementation of breastfeeding policies [34, 35, 39, 41, 43, 45–50, 57], particularly at peripheral health facilities. For example, a study in Ghana found that although almost all policy makers and implementers were aware of the national breastfeeding policy, there was a lack of written guidelines and posters at peripheral health facilities because materials were passed from national to regional, district and then health facilities with bureaucracy and transport barriers encountered between each level [50]. Additionally, policies and guidelines may not be translated into local languages commonly spoken in hospital catchment areas. Poor dissemination was an issue with changing infant feeding guidelines for HIV-positive mothers. A study from South Africa found that the phasing out of free formula for HIV-positive mothers was not clearly explained to health workers particularly at the grassroots level, which led to inconsistent messaging to HIV-positive mothers [43]. Challenges with policy dissemination and implementation were compounded by lack of funding and inadequate staffing and training policies that led to an inability to sustain skilled staff in maternity wards [30–32, 34, 35, 38, 40–42, 44–48].

Facilitators included committed leadership and supportive supervision, in particular by local management at the health facility [30, 34, 40, 52]. Clear and consistent guidelines with adequate dissemination [30, 54] and implementation of policies such as rooming-in, skin-to-skin, and discouraging formula and/or mixed feeding [30, 35, 43, 47, 58] were helpful in facilitating breastfeeding practices. A major facilitator was policies around staffing allocation and training [31, 33–35, 37, 39–42, 50, 51, 53–56]. This included increasing the number of skilled staff and task-sharing, such as in Malawi where over 600 lay support staff were trained to overcome challenges of short staffing at hospitals [34]. Pre-service training during nursing programs [41, 50], BFHI curricula and materials [31, 40, 53, 55, 56] and hands-on training [34, 37, 39, 41, 54] were highlighted as effective methods for training staff.

### Health worker engagement

Factors related to health worker engagement with facility-based breastfeeding support was described by 55.4% (31 of 56) studies, including 28 studies (50.0%) that described barriers [30, 31, 33–35, 37, 39–51, 57, 59–66] and 12 studies (21.4%) that described facilitators [38, 41, 42, 45, 48, 53, 59, 61, 63–65, 67]. Health workers frequently mentioned staffing shortages and heavy workloads in reducing their capacity to provide adequate breastfeeding counselling and other support [31, 33–35, 37, 40–42, 44, 46, 47, 50]. Additionally, gaps in knowledge and misconceptions among health workers led to delivery of inconsistent messaging, specifically around formula feeding [30, 43], pre-lacteal feeds [35, 57, 59], breastfeeding after caesarean delivery [51], that an infant needs to rest after childbirth [50] or mistakenly believed that skin-to-skin contact would increase the risk of hypothermia [63]. A study in Nigeria found that while nursing staff were knowledgeable, non-medical staff frequently gave pre-lacteal feeds [59]. There was considerable misinformation among health workers around infant feeding options for HIV-positive mothers [30, 31, 43, 45, 46, 60, 62, 64–66]. Poor health worker attitudes or willingness to deliver breastfeeding support and a lack of respectful maternity care were also reported barriers [37, 48, 60, 61]. For example, researchers shared how a woman recalled, *“[the nurse] yelled at me, she even came to me and pulled my nipple telling me that I’m failing to breastfeed the baby. She told me to put my breast in baby’s mouth. I would put it. I would say, there is nothing coming out. She said, there is no such thing”* [61]. Another woman shared, *“[The nurses] never helped me. They called me isigqala. I’m like that cow called isigqala, which means I do not have milk …*” [61]. Poor practical skills among health workers were also reported, especially around positioning and attachment and complications management [40, 41, 47, 57, 65].

In general, good knowledge among health workers about breastfeeding benefits and practices was highlighted as a facilitator of facility-based breastfeeding [45, 48, 53, 65, 67]. Specific topics included the management of complications and specialized breastfeeding care and knowledge around infant feeding for HIV-positive mothers. Positive attitudes among health workers and willingness for breastfeeding support [41, 42, 48, 53, 59, 63, 64, 67] as well as providing demonstrations and following up on breastfeeding after counselling [38, 48, 53, 61, 64] were also highly reported facilitators. Providing respectful maternal care and a positive work culture where supporting breastfeeding was a social norm was helpful [38, 64, 65].

### Caregiver engagement

Caregiver factors were mentioned by 75.0% (42 of 56) of studies, including 37 studies (66.1%) that described barriers [31, 32, 34–36, 38, 43, 46, 50–52, 55, 56, 58–62, 64, 66, 68–84] and 27 studies (48.2%) that described facilitators [32, 37, 38, 43, 44, 46, 48, 49, 51, 55, 58, 60, 61, 68, 69, 71–76, 79–82, 84, 85]. Frequently mentioned barriers included inadequate lactation counselling [32, 34, 43, 52, 72, 79] and misconceptions [34–36, 50, 52, 55, 56, 60, 62, 68, 72, 74, 77, 79, 83], such as breastmilk alone would not meet nutritional needs for the baby, formula was healthier, colostrum was dirty or not real breastmilk, infants required water in hot weather or infants required rest after delivery. Peer pressure by relatives and lack of decision-making power was frequently mentioned as a barrier to effective facility-based breastfeeding [36, 46, 52, 56, 60–62, 68, 79, 80]. When counselling was provided, mothers were engaged directly while grandmothers and fathers were rarely included but reported to be influential regarding infant care. A frequently mentioned barrier was fear of HIV transmission and issues around stigma and disclosure of HIV status [31, 36, 46, 52, 55, 62, 66, 70, 73, 74, 79, 80, 84]. A study from Uganda found that maternal HIV-positive status was associated with twice the odds of delayed initiation to breastfeeding (aOR 2.3; 95% CI 1.3–4.2) and reported that the fear of transmission led to hesitancy, even when the mother was counselled and intended to breastfeed [52]. A recent study from Zimbabwe found a prevalent belief that breastmilk from an HIV-positive mother was unsafe for her infant [36]. A study from Malawi suggested that HIV-positive mothers saw exclusive breastfeeding as very demanding on their bodies and made them prone to develop AIDS faster [74]. Caesarean section and breast complications were also frequently mentioned challenges [34, 38, 51, 52, 71, 72, 78, 83]. A study from Uganda found that caesarean delivery was associated with an over 8-fold rate of delayed initiation to breastfeeding (aOR 8.6, 95% CI: 4.7–16.0) [52]. Two studies highlighted the need for specialized breastfeeding support for preterm or low birthweight infants [38, 69], with a study from Tanzania quoting one mother who said, “*I am used to breastfeeding, but not this small baby*” [38].

Previous knowledge about breastfeeding, such as learned through antenatal care [37, 46, 61, 69, 71, 72, 76, 80], and positive attitudes [32, 55, 71, 74, 76, 80–82] helped to facilitate breastfeeding practice. However, the value of receiving postpartum counselling and support to learn breastfeeding skills and techniques was also frequently mentioned [37, 44, 48, 51, 58, 61, 71–73, 75, 76, 82]. This was especially true for preterm and low birthweight babies [38, 48]. Family support was also described as a facilitator [38, 44, 79–81, 84]. For example, a study with first-time mothers in Nigeria found that those with birth companions had significantly earlier initiation of breastfeeding compared to controls without birth companions (*p* < 0.01) [44]. For HIV-positive mothers in particular, it was helpful to have peer support and see other HIV-positive women breastfeeding [46, 60, 79, 84].

A few studies mentioned maternal characteristics including parity, education, age, marital status and other indicators of socio-economic status such as private hospital attendance, house ownership and income as factors influencing breastfeeding. However, it was highly contextual to local settings and there were many inconsistencies between barriers [69, 81, 83] and facilitators [49, 68, 81, 82, 85].

Table 2 summarizes the barriers and facilitators to facility-based breastfeeding in SSA. Table S6 includes the barriers and facilitators reported in each study and Table S7 includes a breakdown of themes summarized by studies.

**Table 2:** Barriers and facilitators to facility-based breastfeeding support in SSA

	Barriers	Facilitators
Health facilities infrastructure and supplies	• Overcrowding and lack of space • Lack of privacy or quiet place to breastfeed • Insufficient equipment or supplies	• Supplies that support breastfeeding practice
Supportive policies and policy implementation	• Poor leadership and management structures • Lack of guidelines/policies or their limited implementation • Inability to sustain skilled staff with due to staffing and training policies	• Commitment and leadership • Mechanisms of regulation and supportive supervision • Clear and consistent guidelines with adequate dissemination and policy implementation • Adequate training and staffing policies and allocation
Health worker engagement	• Staffing shortages and workload • Gaps in knowledge, misconceptions and inconsistent messaging • Gaps in practical skills and management of complications • Poor health worker attitude or willingness • Poor respectful maternity care	• Good knowledge among health workers about breastfeeding benefits and practices • Positive attitudes and willingness • Providing demonstrations and following up • Providing respectful maternal care • Positive work culture and social norms among medical staff supporting breastfeeding
Caregiver engagement	• Gaps in knowledge • Misconceptions, beliefs and cultural practices • Fear of HIV transmission or stigma • Difficulty with breastfeeding practice and receiving inadequate health worker support • Insufficient milk production • Health conditions of mother/infant • Insufficient milk production • Maternal characteristics	• Acceptability and knowledge • Received postpartum health worker counselling and/or support • Learning skills and techniques to improve breastfeeding practice • Supportive social networks and peer support groups: HIV+ peers • Absence of breast problems • Maternal characteristics

## Discussion

The purpose of this review was to compile facility-based barriers and facilitators to early and exclusive breastfeeding in SSA. Relatively few studies described the effect of facility-level infrastructure and supply factors on breastfeeding while caregiver factors were frequently described, particularly around knowledge and attitudes. The focus on counselling to provide adequate education, dispel misconceptions and give support to the mother and her family highlights the importance of respectful care. Another broad area of focus was on the implementation of policies and guidelines, which were often available but their implementation required staffing to deliver, commitment by health management to prioritize and monitor, and coordination between different health system levels.

Other reviews also found an emphasis on health worker and caregiver breastfeeding knowledge, skills and perceptions. Similar to our findings, enhanced knowledge and positive perceptions of breastfeeding supported practice, while negative attitudes and inaccurate knowledge regarding exclusive breastfeeding diminished breastfeeding rates [18–22]. Many studies evaluate interventions to improve maternal and health worker breastfeeding knowledge [20–22, 86–90]. However, our focus on facility-based factors also highlighted the need to examine infrastructural gaps, which led to overcrowding challenges and lack of privacy to counsel, the latter of which was critical for HIV-positive mothers in particular. In LMICs, home intervention alone appeared more effective than hospital intervention alone [88] while the same was not the case in high-income countries [21]. Taken in consideration of our review’s findings, this may indicate gaps in facility-based breastfeeding support in LMICs, for example in insufficient space and health worker time to adequately counsel mothers and influential family members, as well as in trained health workers to address breastfeeding challenges.

The finding that caesarean section delivery negatively impacted early breastfeeding practice was supported by five reviews [7, 20, 22, 87, 91]. This could be due to exhaustion, decreased lactogenesis or an intent not to breastfeed among some mothers [20]. Another review also cited breast complications, low birthweight and prematurity as barriers to breastfeeding initiation and continuation, a finding that supports similar results in our review. While briefly mentioned in previous reviews, caesarean section, premature infants and HIV-positive status were particularly emphasized in our review that focused on facility-based factors. The emphasis on HIV status also highlights the importance of context as it emerged as a critical factor in SSA settings while not mentioned in other general reviews.

### Implications for policy and practice

BFHI emphasizes the important role hospitals play in promoting breastfeeding. However, this needs engagement of staff, adequate space and contextualization to local needs. Within the SSA context, findings from our review highlight the following:

1. Health facility infrastructure and supplies appears to be a neglected area of focus in the promotion of breastfeeding. BFHI policies of rooming-in and immediate skin-to-skin contact require space, which is challenged by overcrowding as facility births increase. In SSA, rates of facility birth have increased by 85% in recent Demographic Health Surveys conducted since 2010 compared to surveys from the 1990s [92]. Adequate staffing and facilities are required to deliver effective breastfeeding counselling and support.
2. There is a need to move beyond the focus on information provision to considering how information is delivered and strengthening respectful maternity care. Health worker engagement is essential to breastfeeding initiation and to provide the necessary support and advice to encourage mothers to continue breastfeeding [17]. As some studies reported verbal abuse, gaps in providing dignified care compromises caregiver engagement. Strengthening health worker communication skills is an area of further exploration and capacity building.
3. Within the SSA context, breastfeeding must be considered in the context of high rates of HIV where there are concerns of transmission. A previous review found that HIV-positive mothers are less likely to adopt exclusive breastfeeding for fear of transmission, cultural beliefs and confusion over infant feeding guidelines [93]. The current review found confusion over changes in infant feeding guidelines and a need for coordination between different health system levels for consistent messaging. Additionally, inadequate protection of mothers’ confidentiality and lack of decision-making power lowers the openness and comfort of HIV-positive mothers in particular.
4. Though the International Code of Marketing of Breastmilk Substitutes was implemented in 1981, its implementation and monitoring falls under the responsibility of local governments [94], which remains a challenge within SSA health facilities. For example, infant formula promoters advocating formula use to health workers were documented in Niger [35] while overall compliance with the Code was reported around 54% at BFHI hospitals in Ghana due to attrition of trained staff along with inadequate in-service training for new staff and poor regional and national monitoring [47]. The current review found evidence of misconceptions around formula use from both health workers and caregivers and inconsistent messaging around formula use, particularly associated with concern for HIV transmission [45].

### Strengths and weaknesses

To the best of our knowledge, our review is the first to focus on facility-based barriers and facilitators to early and exclusive breastfeeding in SSA and is strengthened by a comprehensive and systematic search process informed by pediatric experts from Africa. Studies from numerous countries across Africa and over two-thirds of articles rated as good or fair quality lends to completeness and validity of review findings. Limitations of the review include the restriction to English-text articles, which biases against research from French-speaking countries in Africa, and the ambiguous boundary between facility- and community-based factors. While our review aimed to illuminate factors that are modifiable at the facility-level, we acknowledge that the linkages between community and health facilities is also an important area of strengthening and facility- and community-based factors can overlap.

## Conclusion

A key goal of the WHO Global Nutrition Targets 2025 is to increase the rate of exclusive breastfeeding in the first 6 months of life to 50% to support achieving the Sustainable Development Goals 2 to end hunger and 3 to ensure healthy lives. Increased breastfeeding rates can contribute to the reduction of child mortality disparities in SSA and provide equal opportunity for all children to grow and thrive. As rates of facility births dramatically rise in SSA, health facilities are key spaces to promote optimal breastfeeding practices. Our review of facility-based barriers and facilitators of early and exclusive breastfeeding support in SSA highlight that it is critical to strengthen capacities in respectful maternity care and ensure appropriate spaces and adequate staff training to support specialized care for vulnerable groups, such as HIV-positive mothers and preterm infants.

## Supplementary Information

**Additional file 1:** **Table S1.** PRISMA checklist.

**Additional file 2:** **Table S2.** Search strategy.

**Additional file 3:**: **Table S3.** Excluded studies.

**Additional file 4:** **Table S4.** Characteristics of included studies.

**Additional file 5:** **Table S5.** Quality assessment of included studies.

**Additional file 6 **: **Table S6.** Barriers and facilitators to facility-based breastfeeding support in Sub-Saharan Africa reported in each study.

**Additional file 7:** **Table S7.** Summary of themes by studies.

## Data Availability

All data generated or analysed during this study are included in this published article and its. supplementary information files. Table S1 – PRISMA checklist. Table S2 - Search strategy. Table S3 - Excluded studies. Table S4 - Characteristics of included studies. Table S5 - Quality assessment of included studies. Table S6 - Barriers and facilitators to facility-based breastfeeding support in Sub-Saharan Africa reported in each study. Table S7 - Summary of themes by studies.

## Abbreviations

BFHI: Baby-Friendly Hospital Initiative
LMICs: Low- and middle-income countries NIH National Institutes of Health
PRISMA: Preferred Reporting Items for Systematic Reviews and Meta-Analysis
SSA: Sub-Saharan Africa
WHO: World Health Organization
