# Supplementary material for: Barriers and facilitators for early and exclusive breastfeeding in health facilities in Sub-Saharan Africa: a systematic review
Source: Glob Health Res Policy. 2021 Jul 6;6:21. doi: 10.1186/s41256-021-00206-2 (PMC8259208; doi:10.1186/s41256-021-00206-2)
Supplement: Supplementary file 2 — Additional file 2: Table S2. Search strategy. [file 41256_2021_206_MOESM2_ESM.docx]

**Table S2 – Search strategy**

| Intervention | “breast feeding” or breastfeed* or breast feed* or breastfed* or breast-fed* or breast-feed* or “breast milk” or breastmilk or breast-milk or “exclusive breastfeeding support” or “baby friendly hospital initiative” or “BFHI” |
| --- | --- |
| Context | Africa or sub-Sahar* or south* Africa or west* Africa or east* Africa or Angola or Benin or Botswana or Burkina Faso or Burkina Fasso or Burundi or Cameroon or Cameroons or Cameron or Camerons or Cape Verde or Cabo Verde or Central African Republic or Chad or Comoros or Comoro Islands or Comores or Mayotte or Congo or Zaire or Cote d'Ivoire or Ivory Coast or Djibouti or French Somaliland or Eritrea or Estonia or Ethiopia or Gabon or Gabonese Republic or Gambia or Ghana or Guinea or Guinea-Bissau or Kenya or Lesotho or Basutoland or Liberia or Madagascar or Malawi or Nyasaland or Mali or Mauritania or Mauritius or Mozambique or Namibia or Niger or Nigeria or Rwanda or Ruanda or “Sao Tome and Principe” or Senegal or Seychelles or Sierra Leone or Somalia or South Africa or Sudan or South Sudan or Swaziland or Eswatini or Tanzania or Togo or Uganda or Zambia or Zimbabwe or Rhodesia  hospital* or clinic* or "referral hospital" or "tertiary hospital" or "district hospital" or "health centre" or "health center" or "health facility" or "health facilities" |
| Outcome | Barrier* or challenge* or delay* or facilitator* or enabler* or implement* or factor* or determinant* or program* or “implementation science” or “implementation research” or “health services research |
