# Supplementary material for: Barriers and facilitators for early and exclusive breastfeeding in health facilities in Sub-Saharan Africa: a systematic review
Source: Glob Health Res Policy. 2021 Jul 6;6:21. doi: 10.1186/s41256-021-00206-2 (PMC8259208; doi:10.1186/s41256-021-00206-2)
Supplement: Supplementary file 3 — Additional file 3:: Table S3. Excluded studies. [file 41256_2021_206_MOESM3_ESM.docx]

**Table S3: Excluded studies**

| **Reference** | **Title** | **Reason for exclusion** |
| --- | --- | --- |
| Ahmed et al 2019 | Trends and determinants of early initiation of breastfeeding and exclusive breastfeeding in Ethiopia from 2000 to 2016 | Found that facility delivery was associated with exclusive breastfeeding practice but lack of information on facility-based barriers and facilitators |
| Ahmed et al 2019 (2) | Trends and determinants of early initiation of breastfeeding and exclusive breastfeeding in Ethiopia from 2000 to 2016. | Duplicate, see entry above |
| Belachew 2019 | Timely initiation of breastfeeding and associated factors among mothers of infants age 0-6months old in Bahir Dar City, Northwest, Ethiopia, 2017: a community based cross-sectional study | Found that facility delivery was associated with exclusive breastfeeding practice but lack of information on facility-based barriers and facilitators |
| Benjamin et al 2019 | Knowledge of the importance of exclusive breastfeeding among lactating young mothers in Enugu Urban, Nigeria | Not enough information on facility-based barriers and facilitators |
| Berde 2018 | Factors Associated with Bottle Feeding in Namibia: Findings from Namibia 2013 Demographic and Health Survey. | Found that facility delivery was associated with exclusive breastfeeding practice but lack of information on facility-based barriers and facilitators |
| Bergamaschi et al 2019 | Is childbirth location associated with higher rates of favourable early breastfeeding practices in Sub-Saharan Africa? | Found that facility delivery was associated with early breastfeeding practice but lack of information on facility-based barriers and facilitators |
| Borstlap et al 2016 | Do pregnant women know how to correct inverted nipples? | Did not target our study population |
| Cherop et al 2009 | Barriers To Exclusive Breastfeeding Among Infants Aged 0-6 Months In Eldoret Municipality, Kenya | Community based factors and did not explore facility-based barriers and facilitators |
| Chintalapudi et al 2018 | Infant and Young Child Feeding Decision Making and Practices: Malawian Mothers' and Fathers' Roles in the Context of HIV. | Lack of information on facility-based barriers and facilitators |
| Chipojola et al 2019 | Determinants of breastfeeding practices among mothers in Malawi: a population-based survey | Found that facility delivery was associated with exclusive breastfeeding practice but lack of information on facility-based barriers and facilitators |
| Ekubay et al 2018 | Initiation of breastfeeding within one hour of birth among mothers with infants younger than or equal to 6 months of age attending public health institutions in Addis Ababa, Ethiopia. | Found that facility delivery was associated with exclusive breastfeeding practice but lack of information on facility-based barriers and facilitators |
| Ferguson et al 2006 | A process evaluation of nurses' implementation of an infant feeding counseling protocol for HIV-infected mothers: the Breastfeeding, Antiretroviral and Nutrition (BAN) Study in Lilongwe, Malawi. | To be combined with the published article, "Evaluating nurses' implementation of an infant-feeding counseling protocol for HIV-infected mothers: the BAN Study in Lilongwe, Malawi." Ferguson et al 2009 |
| Ganle et al 2019 | Discontinuation of Exclusive Breastfeeding in Ghana: A Longitudinal, One-Group Observational Study of Postnatal Mothers With Children 0-6 Months old. | Did not discuss facility-related barriers and facilitators |
| Horwood et al 2019 | An educational intervention to update health workers about HIV and infant feeding | Unclear about breastfeeding scope – early, exclusive or complementary |
| Jama et al 2018 | Autonomy and infant feeding decision-making among teenage mothers in a rural and urban setting in KwaZulu-Natal, South Africa. | Community based factors and did not explore facility-based barriers and facilitators |
| Kinshella et al 2020 | “So sometimes, it looks like it’s a neglected ward”: Health worker perspectives on implementing kangaroo mother care in southern Malawi | Does not discuss facility-based breastfeeding barriers and facilitators |
| Lakati et al 2010 | The effect of pre-lacteal feeding on full breastfeeding in Nairobi, Kenya. | Not enough detail on facility-based barriers and facilitators |
| Lawal et al 2017 | Examining maternal age, breastfeeding self-efficacy and health locus of control in psychological wellbeing of mothers. | Unclear on exclusive or complementary breastfeeding |
| Magowan et al 2020 | Exploring the barriers and facilitators to the acceptability of donor human milk in eastern Uganda – a qualitative study | Elicited caregiver perspectives and acceptability of donor human milk but not enough detail on facility-based barriers and facilitators |
| Maree et al 2017 | Quality Improvement Initiative for Family-Centered Care in the Neonatal Intensive Care Unit of a Tertiary Hospital in South Africa. | Could not isolate the intervention for breastfeeding |
| Minas and Ganga- Limando 2016 | Social-Cognitive Predictors of Exclusive Breastfeeding among Primiparous Mothers in Addis Ababa, Ethiopia. | Does not discuss facility-based breastfeeding barriers and facilitators |
| Minas and Ganga-Limando 2016 | Impact of knowledge and attitude of primiparous women in Addis Ababa, Ethiopia on achieving optimal breastfeeding practices | Lack of information on facility-based barriers and facilitators |
| Nankumbi et al 2019 | Predictors of breastfeeding self-efficacy among women attending an urban postnatal clinic, Uganda | Not enough information on facility-based barriers and facilitators; does not directly mention how supports increased the self-efficacy of these mothers |
| Nkoka et al 2019 | Determinants of timely initiation of breast milk and exclusive breastfeeding in Malawi: a population-based cross-sectional study | Found that facility delivery was associated with exclusive breastfeeding practice but lack of information on facility-based barriers and facilitators |
| Nyade et al 2016 | Beliefs about Supporting Mothers to Exclusively Breastfeed for 6 Months: An Elicitation Study of Health Professionals Working in Maternal-Child Health Clinics in Nairobi, Kenya. | Duplicate that made it to full text review |
| Okanda et al 2018 | Higher likelihood of 6-months exclusive breastfeeding among HIV infected than uninfected mothers: a household survey in Kenya. | Lack of information on facility-based barriers and facilitators |
| Sadoh et al 2009 | Experiences of HIV positive mothers who chose not to breastfeed their babies in Nigeria. | Did not discuss facility-related barriers and facilitators |
| Seonandan et al 2016 | A review of infant and young child feeding practice in hospital and the home in KwaZulu-Natal Midlands. | While infants under 6 months were reported as a separated age group, the results do not elaborate in depth on facility based barriers for that age group (just say that "in actual practice, infants less than 6 months of age are able to breastfeed on demand only in one third of facilities" but does not report reasons why). |
| Siziba et al 2015 | Low rates of exclusive breastfeeding are still evident in four South African provinces | Not enough information on facility-based barriers and facilitators |
| Soti-Ulberg et al 2020 | Scaling up breastfeeding policy and programs in Samoa: application of the Becoming Breastfeeding Friendly initiative | Not in Africa |
| Tahsina et al 2020 | Immediate newborn care and breastfeeding: EN-BIRTH multi-country validation study | Evaluated validity of early initiation to breastfeeding measurement but could not isolate breastfeeding facility-based barriers and facilitators from other immediate newborn care practices |
| Umeobieri et al 2018 | Perception and practice of breastfeeding among HIV positive mothers receiving care for prevention of mother to child transmission in South-East, Nigeria. | Not enough information on facility-based barriers and facilitators |
| Visser et al 2016 | Breastfeeding among mothers in the public health sector: the role of the occupational therapist | Unclear on exclusive or complementary breastfeeding |
| Wainaina et al 2018 | Exploring the Experiences of Middle Income Mothers in Practicing Exclusive Breastfeeding in Nairobi, Kenya. | Not enough detail on facility-based barriers and facilitator |
| Welch et al 2019 | A Bottleneck Analysis of Care and Feeding of the Small and Sick Newborn in Malawi: Findings and Proposed Solutions | Conference proceedings |
| Williams et al 2016 | Breastfeeding and Complementary Feeding Practices among HIV-Exposed Infants in Coastal Tanzania | Found that facility delivery was associated with exclusive breastfeeding practice but lack of information on facility-based barriers and facilitators |
| Woldeamanuel 2020 | Trends and factors associated to early initiation of breastfeeding, exclusive breastfeeding and duration of breastfeeding in Ethiopia: evidence from the Ethiopia Demographic and Health Survey 2016 | Found that facility delivery was associated with exclusive breastfeeding practice but lack of information on facility-based barriers and facilitators |
