# Supplementary material for: Barriers and facilitators for early and exclusive breastfeeding in health facilities in Sub-Saharan Africa: a systematic review
Source: Glob Health Res Policy. 2021 Jul 6;6:21. doi: 10.1186/s41256-021-00206-2 (PMC8259208; doi:10.1186/s41256-021-00206-2)
Supplement: Supplementary file 4 — Additional file 4: Table S4. Characteristics of included studies. [file 41256_2021_206_MOESM4_ESM.docx]

**Table S4: Characteristics of included studies**

| **Reference** | **Study design and method** | **Country** | **Health facility level** | **Government or private** | **Sample** | **Breastfeeding practice (rate where applicable)** |
| --- | --- | --- | --- | --- | --- | --- |
| Agbozo et al 2019 | Cross-sectional facility assessment (UNICEF/WHO BFHI External Reassessment Tool) and interviews | Ghana | General hospital (secondary-level facility) | Government | 1 BHFI facility; 60 clinical staff, 40 pregnant women, 60 postpartum mothers | Early initiation, exclusive breastfeeding |
| Aghaji 2002 | Cross-sectional patient survey (interviewer administered) | Nigeria | Mixed (health facilities and hospitals) | Mixed | 5 BHFI facilities; 235 mother-infant pairs | Exclusive breastfeeding (33.2%, 78 of 235) |
| Akuse and Obinya 2002 | Cross-sectional healthcare worker survey | Nigeria | Mixed (primary, secondary and tertiary facilities) | Mixed | 24 facilities; 747 healthcare workers (doctors, nurses, lay health workers) | Early initiation |
| Amadhila and Rensburg 2020 | Qualitative explorative design, in-depth interviews | Namibia | Mixed (all hospitals in Namibia) | Mixed | 34 BFHI facilities; 33 nurse managers in charge of baby and mother friendly hospitals | Early initiation, exclusive breastfeeding |
| Amsalu et al 2019 | Observation checklist (WHO Managing Complications in Pregnancy and Childbirth Guide) and follow-up interviews (7-9 days postpartum) | Somalia | Primary health centre | Government | 4 facilities in conflict areas; 253 pregnant women | Early initiation (30.1%, 74 of 246 live births) |
| Awi and Alikor 2006 | Cross-sectional patient survey (WHO/UNICEF BFHI hospital self-appraisal tool) | Nigeria | Teaching hospital (tertiary-level facility) | Government | 1 facility, 500 mother-infant pairs | Early initiation (vaginal delivery: 33.6%, 88 of 262; caesarean delivery: 0.0%, 0 of 238) |
| Chabeda et al 2020 | Qualitative explorative design, in-depth interviews | Kenya | District general hospitals (secondary-level facilities) | Government | 3 facilities; 25 healthcare workers (medical officers, clinical officers, nurses, nutritionists) and breastfeeding peer supporters | Exclusive breastfeeding |
| Chale et al 2016 | Cross-sectional facility assessment (observation checklist based on WHO/UNICEF Baby Friendly Hospital Initiative guidelines) and interviews | Tanzania | Mixed (primary, secondary and tertiary facilities) | Not reported | 11 facilities; 220 healthcare workers (clinicians, nurses) | Exclusive breastfeeding |
| Chaponda et al 2017 | Qualitative explorative design, in-depth interviews | South Africa | Provincial tertiary hospital | Government | 1 facility; 30 postnatal HIV+ mothers | Exclusive breastfeeding |
| Daniels and Jackson 2011 | Cross-sectional facility assessment (The UNICEF/WHO BFHI External Reassessment Tool) and interviews | South Africa | Primary care maternity obstetric units | Not reported | 8 facilities; 8 managers and 45 nurses | Early initiation, exclusive breastfeeding |
| Degefa et al 2019 | Cross-sectional observational checklist (WHO breastfeeding observation criteria) and follow-up structured interview (on discharge) | Ethiopia | Mixed (primary health centre and hospital) | Government | 2 facilities; 252 mother-infant pairs | Early initiation |
| Doherty et al 2019 | Qualitative explorative design, focus group discussions | South Africa | Primary care clinic | Not reported | 12 facilities; 67 mothers with infants <6 months | Early initiation, exclusive breastfeeding |
| Dubik et al 2021 | Cross-sectional healthcare worker survey (developed from the WHO infant and young feeding guide) | Ghana | Primary care clinics | Not reported | 14 facilities; 104 nurses and midwives | Early initiation, exclusive breastfeeding |
| Fadupin et al 2020 | Cross-sectional patient survey (interviewer administered) | Nigeria | Primary health centres | Mixed | Unclear number of facilities; 360 postpartum mothers | Early initiation (40.0%, 144 of 360) |
| Ferguson et al 2009 | Process evaluation (protocol developed from WHO Breastfeeding Counseling Training Manual) with direct observations and interviews | Malawi | Study clinic at district hospital (secondary-level facility) | Government (district hospital) | 1 facility; 6 nurses, 30 HIV+ antepartum and 93 HIV+ postpartum mothers | Exclusive breastfeeding |
| Getnet et al 2020 | Cross-sectional patient survey (interviewer administered) | Ethiopia | General hospitals (secondary-level facility) | Government | 8 hospitals; 349 mothers who delivered by caesarean section | Early initiation (51.9%, 181 of 349) |
| Gejo et al 2019 | Cross-sectional patient survey (interviewer administered) | Ethiopia | General hospitals (secondary-level facility) | Government | 4 facilities; 304 HIV+ mothers attending ART or PMTCT clinics | Exclusive breastfeeding (88.8%, 270 of 304), early initiation (92.1%, 280 of 304) |
| Hasselberg et al 2016 | Qualitative explorative design, semi-structured interviews | Tanzania | Regional referral hospital (secondary-level facility) | Government | 1 facility, 10 mothers with preterm babies, 5 nurses | Exclusive breastfeeding, early initiation |
| Ighogboja et al 1996 | Qualitative explorative design, semi-structured interviews | Nigeria | Teaching hospital (tertiary-level facility) | Government | 1 BFHI facility; 800 mothers | Exclusive breastfeeding (6%, 51 of 800, for first three months), early initiation (55%, 439 of 800, within 24 hours) |
| Iliyasu et al 2019 | Cross-sectional patient survey (interviewer administered) | Nigeria | Teaching hospital (tertiary-level facility) | Government | 1 facility; 203 HIV+ mothers | Exclusive breastfeeding (68.5%, 139 of 203) |
| Kafulafula et al 2014 | Qualitative explorative design, in-depth interviews and focus group discussions | Malawi | Teaching hospital (tertiary-level facility) | Government | 1 facility, 16 HIV + breastfeeding mothers, 5 nurse-midwives and 11 women unknown HIV status | Exclusive breastfeeding |
| Kahindi et al 2020 | Qualitative explorative design, in-depth interviews | Kenya | County hospital (secondary-level facility) | Government | 1 facility; 20 mothers | Exclusive breastfeeding (80% at discharge, 16 of 20) |
| Kalisa et al 2015 | Cross-sectional patient survey (interviewer administered) and focus group discussions | Uganda | Teaching hospital (tertiary-level facility) | Government | 1 facility; 665 mother-infant pairs within 24 hrs following delivery | Early initiation |
| Kassa et al 2021 | Cross-sectional patient survey (interviewer administered) | Ethiopia | General hospitals (secondary-level facility) | Government | 8 hospitals; 348 mothers who delivered vaginally | Early initiation (88.2%, 307 of 348) |
| Kavle et al 2019 | Process evaluation (documentation review, tracking programme data and routine monitoring indicators, self and external facility assessments) | Malawi | Mixed (district health facilities - secondary and primary facilities) | Not reported | 54 BFHI health facilities | Early initiation, exclusive breastfeeding |
| Kusi-Amponsah Diji et al 2017 | Cross-sectional patient survey (interviewer administered) | Ghana | Secondary-level hospital | Government | 1 facility; 240 mothers with infants 3-9 months old | Exclusive breastfeeding (66.7%, 160 of 240) |
| Lang'at et al 2018 | Qualitative explorative design, key informant interviews and focus group discussions | Kenya | Rural referral hospital (secondary-level facility) | Private non-profit (mission hospital) | 1 facility; 35 mothers and 11 healthcare workers (nutritionists, nurses, clinicians and peer workers) | Exclusive breastfeeding |
| Mgolozeli et al 2019 | Cross-sectional healthcare worker survey (based on WHO/UNICEF BFHI hospital self-appraisal tool, UNICEF/WHO 20-h breastfeeding manual) | South Africa | Primary health centres | Government | 18 MBFI facilities; 153 nurses | Exclusive breastfeeding |
| Mohamed et al 2018 | Cross-sectional patient survey (interviewer administered) | Kenya | District hospitals (tertiary-level facility) | Government | 1 facility; 281 mothers with infants 0-5 months old | Early initiation (74.1%, 200 of 281), exclusive breastfeeding (45.5%, 128 of 281) |
| Morgan and Jeggels 2015 | Cross-sectional patient survey (interviewer administered) | South Africa | Maternity-dedicated regional hospital (secondary-level facility) | Government | 1 facility; 100 HIV+ mothers | Exclusive breastfeeding (54%, 54 of 100) |
| Morhason-Bello et al 2009 | Secondary analysis of a randomized controlled trial | Nigeria | Tertiary-level hospital | Government | 1 facility; 94 intervention group (with birth companion) and 115 control group (with no birth companion), all primigravid women | Early initiation (100%, 94 of 94 with birth companion; 41%, 47 of 115 without birth companion by 1 hour postpartum) |
| Moussa et al 2010 | Qualitative exploratory design, non-participatory observation of health workers | Niger | Mixed (hospital, primary health centres) | Government | 3 facilities; 31 healthcare providers (nurses, medical assistants, physicians, pediatricians) | Exclusive breastfeeding, early initiation |
| Mphasha and Skaal 2019 | Cross-sectional healthcare worker survey (based on South African HIV and IYCF guidelines) | South Africa | Primary health care facilities | Government | 21 facilities; 103 nurses | Exclusive breastfeeding |
| Mukerem and Haidar 2012 | Cross-sectional patient survey (interviewer administered) and in-depth interviews | Ethiopia | Mixed (hospital and health centres, government) | Government | 13 facilities with ART and PMTCT services; 384 HIV+ mothers | Early initiation (94.3%, 350 of 371), exclusive breastfeeding (73.0%, 271 of 371) |
| Mukashyaka et al 2020 | Cross-sectional patient survey (interviewer administered) | Rwanda | District hospitals (secondary-level facilities) | Government | 2 facilities; 187 mothers | Early initiation (20.5%, 38 of 187), exclusive breastfeeding (64.2%, 120 of 187) |
| Nabwera et al 2017 | Qualitative exploratory design, non-participatory observation of infant feeding counselling session, focus group discussion, in-depth interview | Kenya | County hospital (secondary-level facility) | Government | 1 facility; hospital and community respondents (overall numbers not reported) | Exclusive breastfeeding |
| Nii Okai Aryeetey and Antwi 2013 | Cross-sectional facility assessment (UNICEF/WHO BFHI External Reassessment Tool) and interviews | Ghana | Mixed (primary, secondary and tertiary facilities) | Government | 6 BFHI facilities; 90 clinical 60 pregnant women, and 150 postpartum women | Exclusive breastfeeding (93.8% at discharge) |
| Nikodem et al 1995 | Cross-sectional hospital survey (UNICEF BFHI hospital self-appraisal tool) and patient survey (World Alliance for Breastfeeding Action) | South Africa | Hospitals (facility level not reported) | Mixed (private and government) | 138 facilities; 516 mothers | Early initiation, exclusive breastfeeding |
| Nyati-Jokomo et al 2019 | Qualitative exploratory design, unstructured in-depth interviews | Zimbabwe | Primary health centre | Government | 2 facilities; 15 postpartum HIV+ mothers with medication adherence challenges | Exclusive breastfeeding |
| Nyawade et al 2016 | Qualitative exploratory design, semi-structured interviews | Kenya | maternal-child health clinics (primary health care) | Government | 6 facilities; 15 healthcare workers | Exclusive breastfeeding |
| Ojofeitimi et al 2000 | Case-controlled design comparing between a BFHI and non-BFHI health facility, patient survey and observation of positioning | Nigeria | Primary health centres | Government | 2 facilities; 217 nursing mothers from BFHI centre, 214 from control non-BFHI centre | Early initiation (70.5% 153 of 217 at BFHI centre; 52.3%, 112 of 214 at non-BFHI centre) exclusive breastfeeding (75.1%, 163 of 217 at BFHI centre; 34.6%, 74 of 214 at non-BFHI centre) |
| Okolo and Ogbonna 2002 | Cross-sectional healthcare worker survey (interviewer administered, based on 10 Steps to Successful Breastfeeding) | Nigeria | Mixed (primary health centre and hospitals) | Private and government | 10 facilities; 250 health workers (doctors, nurses and auxiliary staff) | Early initiation, exclusive breastfeeding |
| Olorunfemi and Dudley 2018 | Cross-sectional patient survey (interviewer administered) | Lesotho | Primary health centres | Government | 191 HIV+ mothers with infants 2 weeks to 6 months old | Exclusive breastfeeding |
| Owoaje et al 2002 | Cross-sectional health worker survey (based on 10 Steps to Successful Breastfeeding and BFHI 18-hour lactation management training course) | Nigeria | Mixed (primary, secondary and tertiary facilities) | Government | 298 nurses in maternal and child health care units | Exclusive breastfeeding |
| Remmert et al 2020 | Cross-sectional postpartum survey (interviewer administered, interview conducted postpartum, using the Duke-UNC Functional Social Support Questionnaire, the UCLA Social Support Inventory, the Internalized AIDS-Related Stigma Scale and a modified Hopkins Symptom Checklist Depression Subscale) | South Africa | District hospital (secondary level facility) | Government | 156 HIV+ mothers enrolled in preventing mother-to-child transmission treatment | Exclusive breastfeeding (28.2%, 44 of 156 ) |
| Senbanjo et al 2014 | Cross-sectional health worker survey and patient survey (interviewer administered for mothers) | Nigeria | Teaching hospital (tertiary-level facility) | Government | 1 facility; 125 clinical staff at the paediatric outpatient clinic and 311 mothers with infants 6-24 months old | Early initiation (27%, 81 of 311), exclusive breastfeeding (36%, 112 of 311) |
| Senghore et al 2018 | Cross-sectional patient survey (interviewer administered) | The Gambia | Teaching hospital (tertiary-level facility) | Government | 1 facility; 334 pregnant women > 4 weeks gestation and postnatal mothers with infants <1 year | Exclusive breastfeeding |
| Shobo et al 2020 | Cross-sectional case observations (assessment tool not described) and interviews | Nigeria | Primary health centres | Government | 10 facilities; 393 mothers observed, 27 mothers interviewed | Early initiation (61%, 240 of 393) |
| Spira et al 2017 | Pre-post study with no control group, facility assessment and interviews | Uganda | Mixed (secondary and tertiary facilities) | Mixed - government and private not-for-profit (mission hospital) | 2 facilities; 4816 women and 157 healthcare workers | Early initiation (8.5%-25.6% pre-post in secondary hospital, 96.8-99.2% pre-post in tertiary hospital) |
| Swarts et al 2010 | Cross-sectional patient survey, focus group discussion | South Africa | District hospital (secondary level facility) | Government | 1 facility; 100 postnatal mothers and 22 mothers with admitted neonates | Exclusive breastfeeding (HIV+ 51%, 19 of 37; HIV- or unknown 85%, 53 of 62) |
| Tawiah-Agyemang et al 2008 | Qualitative exploratory design, semi-structured interviews and focus group discussions | Ghana | Mixed (district hospitals, maternity homes, health posts) | Private and government | 246 recent mothers and 13 health workers, policy makers and implementers | Early initiation |
| Tiruye et al 2018 | Cross-sectional observational checklist (WHO B-R-E-A-S-T Feed observation form) and follow-up patient survey (interviewer administered) | Ethiopia | Mixed (hospital and health centres) | Government | 14 facilities; 422 breastfeeding mothers from EPI and postnatal care units | Effective breastfeeding technique to promote exclusive breastfeeding |
| Tongun et al 2018 | Cross-sectional patient survey (interviewer administered) | South Sudan | Teaching hospital (tertiary-level facility) | Government | 1 facility; 806 mother-infant pairs within 24 hrs of birth | Early initiation (48.1%, 388 of 806 ) |
| van Rensburg et al 2016 | Cross-sectional health worker survey (based on the 2010 WHO Guidelines on HIV and Infant Feeding) | South Africa | Regional hospital (secondary-level facility) | Government | 1 facility; 64 health workers from maternity wards | Exclusive breastfeeding |
| West et al 2019 | Retrospective cohort study, chart review between July 7, 2015 and March 6, 2018 and in-depth interviews | South Africa | Primary health centre | Private non-profit (NGO clinic) | 1 facility; 8116 women (1613 HIV+, 19.9%), interviews with 22 HIV+ mothers, 12 health worker | Exclusive breastfeeding (58% HIV- mothers, 38% HIV+ mothers at 6 months) |
| Yotebieng et al 2015 | Cluster RCT randomly assigned health-care clinics to standard care (control group), BFHI steps 1–9 (steps 1–9 group), or BFHI steps 1–9 plus additional support during well-child visits (steps 1–10 group) | DR Congo | Urban and peri-urban health clinics (facility level not reported) | Private non-profit (mission hospital), government and private for-profit health facilities excluded | 6 facilities; 975 mother–infant pairs | Early initiation (71-76% no significant difference between groups), exclusive breastfeeding (12% control group, 36% in the steps 1–9 group, and 14% in the steps 1–10 group at 6 months) |

*BFHI – Baby Friendly Hospital Initiative; EPI - Expanded Program on Immunization; MBFI - Mother-Baby Friendly Initiative; NGO – non-government organization; RCT – randomized controlled trial; UNICEF - United Nations Children's Fund; WHO – World Health Organization*
