# Supplementary material for: Barriers and facilitators for early and exclusive breastfeeding in health facilities in Sub-Saharan Africa: a systematic review
Source: Glob Health Res Policy. 2021 Jul 6;6:21. doi: 10.1186/s41256-021-00206-2 (PMC8259208; doi:10.1186/s41256-021-00206-2)
Supplement: Supplementary file 5 — Additional file 5: Table S5. Quality assessment of included studies. [file 41256_2021_206_MOESM5_ESM.docx]

**Table S5: Quality assessment of included studies**

**QUALITATIVE** **(CASP Checklist)**

| **Reference** | **1** | **2** | **3** | **4** | **5** | **6** | **7** | **8** | **9** | **10** | **Comments** | **Rating** |
| --- | --- | --- | --- | --- | --- | --- | --- | --- | --- | --- | --- | --- |
| Amadhila and Rensburg 2020 | Y | Y | Y | CD | Y | N | Y | Y | Y | Y | The research objective was clear and appropriate for qualitative design. Participants were nurse managers in charge of the program at the hospitals but it is not explicit if all nurse managers were sampled and if not, how they were selected. Unclear who conducted the interviews and their relationship with the participants; otherwise clear description of data collection and analysis. Policy implications explicitly reported. | Fair |
| Chabeda et al 2020 | Y | Y | Y | Y | Y | CD | CD | N | Y | Y | The research objective was clear and appropriate for qualitative design. Participants purposefully sampled to understand the range of cadres involved in the program. Interviews reportedly conducted by two experienced social scientists but unclear their relationship to the study or with the participants. Ethical approvals obtained but further ethical considerations not elaborated. Limited information on coding and coding framework not provided. To the best of authors’ knowledge, this is the first study of breastfeeding peer supporters in a resource-limited setting. | Fair |
| Chaponda et al 2017 | Y | Y | Y | Y | Y | N | N | N | Y | N | The research objective was clear and appropriate for qualitative design. Participants purposefully sampled to understand local context. Unclear who conducted the interviews and their relationship with the participants, ethical considerations not report, lack of information on qualitative analysis. Policy implications not explicitly reported. | Fair |
| Doherty et al 2019 | Y | Y | Y | Y | Y | N | Y | Y | Y | Y | The research objective to understand mothers’ facility-birth experiences was clear and appropriate for qualitative design. Participants were approached at clinics. Clear description of analysis and discussed data saturation. The study is nested within a larger quasi-experimental study and the influence it may have had on the qualitative responses and the relationship of the qualitative researchers to the larger study is unclear. | Fair |
| Hasselberg et al 2016 | Y | Y | Y | Y | Y | N | Y | Y | Y | Y | The research objective was clear and appropriate for qualitative design. Lead author engaged in participant observation to gain a better understanding of the situation but did not explicitly describe the potential impact this may have had on the research. Clear description of analysis and described methods of rigor. | Fair |
| Ighogboja et al 1996 | N | N | N | Y | N | N | N | N | Y | N | Research objective is unclear. Semi-structured questionnaire was used but unclear the extent of open-ended questions and their analysis. No quotes were provided. Lack of detail on methodology and unclear the implication of findings on BFHI implementation in the hospital. | Poor |
| Kafulafula et al 2014 | Y | Y | Y | Y | Y | CD | Y | Y | Y | Y | Research methodology and analysis clearly described and appropriate to qualitative design and the research question. Ethical considerations with HIV+ mothers taken into account. Relationship between data collectors and interviewers discussed but unclear the relationship between the lead researcher. | Good |
| Kahindi et al 2020 | Y | Y | Y | Y | Y | CD | Y | CD | Y | CD | The research objective was clear and appropriate for qualitative design. Participant selection and recruitment described in detail, with discussion of data saturation. Interviews conducted by experienced local interviewer but unclear their relationship to the study or with the participants. Limited information on coding and coding framework not provided. Policy implications not discussed. | Fair |
| Kalisa et al 2015 | Y | Y | Y | CD | CD | N | Y | N | Y | N | Research question to understand factors associated with delayed initiation of breastfeeding is appropriate for qualitative design in this mixed-methods study. Recruitment process, focus group discussion interview guides and relationship between researcher and participants are unclear. Lack of detail in analysis of qualitative data and data saturation not discussed. Policy implications not discussed. | Poor |
| Lang'at et al 2018 | Y | Y | CD | CD | Y | N | Y | CD | Y | Y | The research objective was clear and appropriate for qualitative design. Reasons for focus group discussion is unclear as the methodology is less appropriate for stigmatized like HIV+, which is discussed in the article. FGDs were held away from the communities to support privacy. Coding framework for analysis described and complemented by illustrative quotes but lack of clarity on the analysis process. | Fair |
| Moussa et al 2010 | Y | Y | Y | CD | Y | N | Y | CD | Y | Y | The research question, qualitative design and ethics pf observational research were clear and well described. However, number of observations/length of fieldwork and the relationship of the first author conducting the observations with the participants is not clear. | Fair |
| Nabwera et al 2017 | Y | Y | Y | CD | Y | N | CD | Y | Y | Y | The research objective was clear and appropriate for qualitative design. Participants were purposively sampled from the hospital and community describe factors from a wide range of perspectives. However, it was not completely clear who were interviewed and composition of the FGDs. The relationship of the first author conducting the observations with the participants is not clear. | Fair |
| Nyati-Jokomo et al 2019 | Y | Y | Y | Y | Y | Y | Y | Y | Y | Y | The research objective was clear and appropriate for qualitative design, which explored women's experiences using unstructured in-depth interviews that emphasized richness over quantity of interviews. Author’s positionality and ethical considerations are well described. A limitation is that the study was conducted at health facilities, which could be intimidating for mothers to express especially the institutional challenges they were encountering but this was intentional to due to high stigma in the community. | Good |
| Nyawade et al 2016 | Y | Y | Y | CD | Y | N | Y | Y | Y | Y | The research objective was clear and appropriate for qualitative design and analysis according to a framework based on the theory of reasoned action was clearly described. Sample was relatively small, a convenience sample and no discussion of data saturation. Relationship between researcher and participants not discussed. | Fair |
| Tawiah-Agyemang et al 2008 | Y | Y | Y | Y | Y | N | N | Y | Y | Y | The research objective was clear and appropriate for qualitative design and analysis was rigorously completed. Declarations state that senior author was funded as a consultant for the Gates Foundation and it is unclear whether that was during this study and its potential influence on relationship with participants. Ethical considerations not discussed. Large sample that triangulated perspectives from policy-makers, health workers and recent mothers. | Fair |

*Y – Yes, N – No, CD – Cannot determine/ cannot tell,*

1. Was there a clear statement of the aims of the research?
2. Is a qualitative methodology appropriate?
3. Was the research design appropriate to address the aims of the research?
4. Was the recruitment strategy appropriate to the aims of the research?
5. Was the data collected in a way that addressed the research issue?
6. Has the relationship between researcher and participant been adequately considered?
7. Have ethical issues been taken into consideration?
8. Was the data analysis sufficiently rigorous?
9. Is there a clear statement of findings?
10. How valuable is the research?

**OBSERVATIONAL COHORT/ CROSS-SECTIONAL SURVEY (NIH Quality Assessment Tool)**

| **Reference** | **1** | **2** | **3** | **4** | **5** | **6** | **7** | **8** | **9** | **10** | **11** | **12** | **13** | **14** | **Comments** | **Rating** |
| --- | --- | --- | --- | --- | --- | --- | --- | --- | --- | --- | --- | --- | --- | --- | --- | --- |
| Agbozo et al 2019 | Y | Y | NR | Y | NR | N | NA | Y | Y | N | Y | NA | NA | N | Cross-sectional study but overall well implemented with triangulation of data between multiple methodologies - hospital record review, observations and interviews | Fair |
| Aghaji 2002 | Y | Y | NR | Y | Y | N | NA | Y | N | N | N | NA | NA | N | Cross-sectional survey where it was unclear if any participants refused to participate. Development of the questionnaire was unclear, self reported breastfeeding outcomes, and not adjusted for potential confounders. | Poor |
| Akuse and Obinya 2002 | Y | Y | Y | Y | N | N | NA | Y | CD | N | NA | NA | NA | N | Cross-sectional survey reportedly using a pretested validated questionnaire but no description of the validation process or citation. | Fair |
| Amsalu et al 2019 | Y | Y | Y | Y | Y | N | Y | Y | Y | N | N | NA | N | Y | Postpartum follow-up time-frame is sufficient for understanding delivery experiences and early initiation to breastfeeding. 76% of eligible women consented but of the 245 women eligible for follow up, 58 were loss to follow up and 11 declined, which is associated with a 28% difference from baseline. | Fair |
| Awi and Alikor 2006 | Y | Y | N | Y | Y | N | NA | Y | Y | N | Y | NA | NA | Y | Cross-sectional survey at a single facility. Lack of clarity on how the 500 consecutive cases were selected from the 1203 deliveries over the nine month period. | Poor |
| Chale et al 2016 | Y | Y | Y | Y | Y | N | NA | Y | Y | Y | N | NA | NA | Y | Cross-sectional study but overall well implemented with triangulation of data between multiple methodologies - hospital record review, observations and interviews. | Fair |
| Daniels and Jackson 2011 | Y | Y | NR | Y | N | N | NA | Y | Y | N | N | NA | NA | N | Cross-sectional study but overall well implemented with triangulation of data between multiple methodologies - hospital record review, observations and interviews. | Fair |
| Degefa et al 2019 | Y | Y | Y | Y | Y | N | Y | Y | Y | N | Y | NA | NA | N | Cross-sectional study that used a standardized observational checklist adopted from the WHO breastfeeding observational form and structured client exit interview that were both pretested. Sample sized based on health facility records and participants selected using a systematic random sampling technique. Triangulation of data between multiple methodologies - hospital record review, observations and interviews. Only descriptive statistics conducted. | Fair |
| Dubik et al 2021 | Y | Y | Y | Y | N | N | Y | N | Y | N | Y | NA | NA | N | A cross-sectional study conducted in multiple facilities with participation of 104 out of 144 eligible participants recruited over a span of four months. Purposeful sampling used; no sample size justification given. Only descriptive statistics conducted. | Poor |
| Fadupin et al 2020 | Y | Y | NR | CD | NR | N | NA | NA | Y | NA | Y | NA | NA | N | A cross-sectional study that randomly selected postpartum mothers for a survey. Process of random selection not described. Refusal rate of eligible participants not reported. Eligibility criteria not explicitly detailed and time period of data collection not specified. Sample size justification not reported. Only descriptive statistics conducted. | Poor |
| Ferguson et al 2009 | Y | N | N | N | N | N | NA | NA | N | N | N | NA | NA | N | A process evaluation of a breastfeeding protocol implementation. A convenience sample of 6 out of 20 nurses for the health worker component of the study highlights critical potential for sampling bias. The breastfeeding protocol specific to the study is not clearly described. | Poor |
| Gejo et al 2019 | Y | Y | Y | Y | Y | N | NA | Y | Y | N | Y | NA | NA | Y | Cross-sectional study with a systematic sampling technique and 100% response rate of eligible women. Use of a pretested, structured questionnaire and adjusted for potential confounders in multivariable logistic regression analyses | Fair |
| Getnet et al 2020 | Y | Y | Y | Y | Y | N | NA | Y | Y | N | Y | NA | NA | Y | Cross-sectional study with a systematic sampling technique and 98% response rate of eligible women. Use of a pretested, structured questionnaire and adjusted for potential confounders in multivariable logistic regression analyses | Fair |
| Iliyasu et al 2019 | Y | Y | NR | Y | Y | N | NA | Y | Y | N | Y | NA | NA | Y | Cross-sectional study with a survey validated for use within the current population in previous studies and pretested within the current study. Evaluation of outcome using systematic, validated system. | Fair |
| Kassa et al 2021 | Y | Y | Y | Y | Y | N | NA | Y | Y | N | Y | NA | NA | Y | Cross-sectional study with a systematic sampling technique and 98% response rate of eligible women. Use of a pretested, structured questionnaire and adjusted for potential confounders in multivariable logistic regression analyses | Fair |
| Kavle et al 2019 | Y | Y | Y | Y | N | N | Y | Y | Y | N | N | NA | N | N | Process evaluation conducted with all BHFI facilities where implemented. Use of routine surveillance indicators and specific program indicators, which were discussed with stakeholders at a two day workshop for contextualization at the end of the two year implementation. | Fair |
| Kusi-Amponsah Diji et al 2017 | Y | Y | N | Y | Y | N | NA | Y | Y | N | Y | NA | NA | Y | Cross-sectional study with pretested structured survey conducted until sample size calculation was satisfied. Women were recruited into the study using a simple random sampling techniques (two opaque envelopes) though it is unclear how many eligible women declined participation. Clear definition of outcome and adjusted for potential confounders. | Fair |
| Mgolozeli et al 2019 | Y | Y | Y | Y | N | N | NA | Y | Y | N | Y | NA | NA | N | Cross-sectional study with a self-administered questionnaire but acceptable high response rate from eligible health workers (85%). Questionnaire was based off of UNICEF/WHO/BFHI documents and was pretested. | Fair |
| Mohamed et al 2018 | Y | Y | NR | Y | Y | N | NA | Y | Y | N | Y | NA | NA | N | Cross-sectional study . Study participants were selected using simple random sampling technique (Table of Random  Numbers) until sample size calculation was fulfilled. Use of a pretested structured questionnaire validated for use within the study population. The rate of refusal among eligible women not reported. Exclusive breastfeeding practice was based on a 24 hour recall may overestimate but is the method recommended by WHO and used in the DHS globally. Descriptive statistics only without adjustment for potential confounders. | Fair |
| Morgan and Jeggels 2015 | Y | Y | Y | Y | N | N | NA | Y | Y | N | Y | NA | NA | N | Cross-sectional study with a structured survey. Sample size was determined to be 100 participants with “as it is a round  Number” for justification. Questionnaire was adapted from an additional study and unclear whether updated version was pretested. Breastfeeding behaviours are self-reported. Descriptive statistics only without adjustment for potential confounders. | Poor |
| Mphasha and Skaal 2019 | Y | Y | Y | Y | Y | N | NA | Y | Y | N | Y | NA | NA | Y | Cross-sectional study conducted at multiple facilities but time frame of data collection is not clear and it is not clear if anyone refused participation or recruitment stopped after reaching sample size. Convenience sampling and analyses did not control for potential confounders. While knowledge could be objectively evaluated through questions based on the guidelines, practice is self-reported and sensitive to recall bias and social desirability bias. | Poor |
| Mukashyaka et al 2020 | Y | Y | NR | Y | Y | N | NA | Y | Y | N | Y | NA | NA | N | A cross-sectional study using a validated questionnaire and pilot tested in local setting. Refusal rate of eligible participants not reported. Breastfeeding behaviours are self-reported. Descriptive statistics only without adjustment for potential confounders. | Poor |
| Mukerem and Haidar 2012 | Y | Y | Y | Y | Y | N | NA | Y | Y | N | N | NA | NA | Y | Cross-sectional mixed methods study primarily focused on the quantitative survey complemented by some qualitative interviews. Because the qualitative interviewees were selected due to their active participation in the quantitative questionnaire, high likelihood of sampling bias. Analyses adjusted for potential confounding factors. | Poor |
| Nii Okai Aryeetey and Antwi 2013 | Y | Y | NR | Y | NR | N | NA | Y | Y | N | Y | NA | NA | N | Cross sectional study but overall well implemented with triangulation of data between multiple methodologies - hospital record review, observations and interviews. | Fair |
| Nikodem et al 1995 | Y | Y | N | N | N | N | NA | CD | Y | N | N | NA | NA | N | A postal questionnaire sent to hospitals and a self-evaluation published in a mother oriented magazine. High likelihood of sampling bias as hospital response rate was low and magazine targets a certain population of mothers. Self reported data with no means of verification. | Poor |
| Okolo and Ogbonna 2002 | Y | Y | Y | Y | N | N | NA | Y | Y | N | Y | NA | NA | N | Cross-sectional study with a pretested structured questionnaire conducted in multiple facilities. Responses were reported separately and combined for health worker position but no adjustment conducted for cadre in final analyses. | Fair |
| Olorunfemi and Dudley 2018 | Y | Y | NR | Y | Y | N | NA | Y | Y | N | Y | NA | NA | N | Cross-sectional study with pretested, structured questionnaire. Refusal rate of eligible participants not reported highlighting potential sampling bias. Process of "random sampling" not described. | Poor |
| Owoaje et al 2002 | Y | Y | Y | Y | N | N | NA | NA | Y | N | Y | NA | NA | Y | Cross-sectional study at multiple health facilities. Clear exposure and outcomes evaluated and multivariate analyses conducted. | Fair |
| Remmert et al 2020 | Y | Y | NR | Y | Y | N | NA | Y | Y | N | N | NA | NA | N | Cross-sectional study with refusal rate of eligible participants not reported, which highlights potential sampling bias. Self-reported breastfeeding practices and no adjustment for potential confounders. | Poor |
| Senbanjo et al 2014 | Y | Y | Y | Y | N | N | NA | Y | Y | N | N | NA | NA | Y | Cross-sectional study where breastfeeding practices were self reported. However, potential confounders were considered. | Fair |
| Senghore et al 2018 | Y | Y | NR | Y | Y | N | NA | Y | Y | N | Y | NA | NA | Y | Cross-sectional study at a single facility over a short period (3 months) limits generalizability. Refusal rates of eligible participants suggests that potential sampling bias. Refusal rates of eligible participants and excluded cases due to missing information or language barrier not reported though raised as an issue in sample size calculations. Exclusion of women with pregnancy complications may bias results. However, did use a validated questionnaire and adjusted for potential confounders. | Poor |
| Shobo et al 2020 | Y | Y | Y | N | NR | N | NA | NA | Y | N | Y | NA | NA | N | Cross-sectional mixed methods study primarily focused on the quantitative observations (assessment tool not described) complemented by some qualitative interviews. Quantitative and qualitative findings came from different groups of women (quantitative recruited in Dec 2017 and qualitative recruited almost a year later in Nov 2018) with no discussion on how this may influence their findings. No potential confounders adjusted. Self report of breastfeeding practices and intentions. | Poor |
| Swarts et al 2010 | Y | Y | NR | N | Y | N | NA | Y | Y | N | N | NA | NA | N | Cross-sectional mixed methods study primarily focused on the quantitative survey complemented by some qualitative interviews. Refusal rate of eligible participants not discussed, suggesting possible sampling bias. Quantitative and qualitative findings came from different groups of women with no discussion on how this may influence their findings. No potential confounders adjusted. Self report of breastfeeding practices and intentions. | Poor |
| Tiruye et al 2018 | Y | Y | Y | Y | Y | N | CD | NA | Y | N | Y | NA | NA | Y | Cross-sectional study conducted in multiple facilities conducted over the span of one month. Responses are not separated between postpartum and EPI unit mothers. Use of observations in addition to questionnaire strengthens results though possible Hawthorne effect in the short observation periods (5 minutes). Lack of a validated WHO instrument though tools were structured and pretested. The study considered potential confounders | Fair |
| Tongun et al 2018 | Y | Y | Y | Y | Y | N | Y | Y | Y | N | N | NA | NA | Y | Cross-sectional study design in a single facility over the span of four months limits generalizability. Exclusion of mothers of ill infants may influence outcome. Overall, a systematically designed and implemented study. | Fair |
| van Rensburg et al 2016 | Y | Y | N | Y | N | N | NA | NA | Y | N | N | NA | NA | NA | A cross-sectional study with less than 50% of participants given questionnaires returned the survey, which indicates a high potential for sampling bias. | Poor |
| West et al 2019 | Y | Y | NR | Y | N | Y | CD | NA | Y | Y | Y | NA | NR | N | Cohort study with limited methodology and reporting from the database review including timeframe of chart review and comprehensiveness of data and loss to follow up. Potential confounding variables not described or controlled for. | Fair |

*Y – Yes, N – No, CD – Cannot determine/ cannot tell, NR – not reported, NA – not applicable*

1. Was the research question or objective in this paper clearly stated?
2. Was the study population clearly specified and defined?
3. Was the participation rate of eligible persons at least 50%?
4. Were all the subjects selected or recruited from the same or similar populations (including the same time period)? Were inclusion and exclusion criteria for being in the study prespecified and applied uniformly to all participants?
5. Was a sample size justification, power description, or variance and effect estimates provided?
6. For the analyses in this paper, were the exposure(s) of interest measured prior to the outcome(s) being measured?
7. Was the timeframe sufficient so that one could reasonably expect to see an association between exposure and outcome if it existed?
8. For exposures that can vary in amount or level, did the study examine different levels of the exposure as related to the outcome (e.g., categories of exposure, or exposure measured as continuous variable)?
9. Were the exposure measures (independent variables) clearly defined, valid, reliable, and implemented consistently across all study participants?
10. Was the exposure(s) assessed more than once over time?
11. Were the outcome measures (dependent variables) clearly defined, valid, reliable, and implemented consistently across all study participants?
12. Were the outcome assessors blinded to the exposure status of participants?
13. Was loss to follow-up after baseline 20% or less?
14. Were key potential confounding variables measured and adjusted statistically for their impact on the relationship between exposure(s) and outcome(s)?

**CASE-CONTROL STUDY (NIH Quality Assessment Tool)**

| **Reference** | **1** | **2** | **3** | **4** | **5** | **6** | **7** | **8** | **9** | **10** | **11** | **12** | **Comments** | **Rating** |
| --- | --- | --- | --- | --- | --- | --- | --- | --- | --- | --- | --- | --- | --- | --- |
| Ojofeitimi et al 2000 | Y | Y | N | Y | Y | Y | NR | NR | Y | Y | NR | N | Comparison of a BFHI designed and undesignated health facility. Main limitation is that the associations were not adjusted to potential confounders. | Fair |

*Y – Yes, N – No, CD – Cannot determine/ cannot tell, NR – not reported, NA – not applicable*

1. Was the research question or objective in this paper clearly stated and appropriate?
2. Was the study population clearly specified and defined?
3. Did the authors include a sample size justification?
4. Were controls selected or recruited from the same or similar population that gave rise to the cases (including the same timeframe)?
5. Were the definitions, inclusion and exclusion criteria, algorithms or processes used to identify or select cases and controls valid, reliable, and implemented consistently across all study participants?
6. Were the cases clearly defined and differentiated from controls?
7. If less than 100 percent of eligible cases and/or controls were selected for the study, were the cases and/or controls randomly selected from those eligible?
8. Was there use of concurrent controls?
9. Were the investigators able to confirm that the exposure/risk occurred prior to the development of the condition or event that defined a participant as a case?
10. Were the measures of exposure/risk clearly defined, valid, reliable, and implemented consistently (including the same time period) across all study participants?
11. Were the assessors of exposure/risk blinded to the case or control status of participants?
12. Were key potential confounding variables measured and adjusted statistically in the analyses? If matching was used, did the investigators account for matching during study analysis?

**BEFORE-AFTER (PRE-POST) STUDIES WITH NO CONTROL GROUP** **(NIH Quality Assessment Tool)**

| **Reference** | **1** | **2** | **3** | **4** | **5** | **6** | **7** | **8** | **9** | **10** | **11** | **12** | **Comments** | **Rating** |
| --- | --- | --- | --- | --- | --- | --- | --- | --- | --- | --- | --- | --- | --- | --- |
| Spira et al 2017 | Y | Y | Y | NR | Y | Y | Y | CD | NA | Y | Y | Y | A pre-post design was used instead an intended time-series design to shorten the study due financial constraints. Analysis of potential confounders and potential secular trends not done. Researchers cautioned that pre-post design tend to overestimate effects. | Fair |

*Y – Yes, N – No, CD – Cannot determine/ cannot tell, NR – not reported, NA – not applicable*

1. Was the study question or objective clearly stated?
2. Were eligibility/selection criteria for the study population prespecified and clearly described?
3. Were the participants in the study representative of those who would be eligible for the test/service/intervention in the general or clinical population of interest?
4. Were all eligible participants that met the prespecified entry criteria enrolled?
5. Was the sample size sufficiently large to provide confidence in the findings?
6. Was the test/service/intervention clearly described and delivered consistently across the study population?
7. Were the outcome measures prespecified, clearly defined, valid, reliable, and assessed consistently across all study participants?
8. Were the people assessing the outcomes blinded to the participants' exposures/interventions?
9. Was the loss to follow-up after baseline 20% or less? Were those lost to follow-up accounted for in the analysis?
10. Did the statistical methods examine changes in outcome measures from before to after the intervention? Were statistical tests done that provided p values for the pre-to-post changes?
11. Were outcome measures of interest taken multiple times before the intervention and multiple times after the intervention (i.e., did they use an interrupted time-series design)?
12. If the intervention was conducted at a group level (e.g., a whole hospital, a community, etc.) did the statistical analysis take into account the use of individual-level data to determine effects at the group level?

**CONTROLLED INTERVENTION STUDIES** **(NIH Quality Assessment Tool)**

| **Reference** | **1** | **2** | **3** | **4** | **5** | **6** | **7** | **8** | **9** | **10** | **11** | **12** | **13** | **14** | **Comments** | **Rating** |
| --- | --- | --- | --- | --- | --- | --- | --- | --- | --- | --- | --- | --- | --- | --- | --- | --- |
| Morhason-Bello et al 2009 | Y | Y | Y | N | Y | N | Y | N | Y | NR | Y | Y | Y | Y | Due to the nature of the intervention of birth companionship, there were limitations to blinding. Midwives were not blinded to treatment allocation thought there was separation on delivery decisions by consultants or senior registrar to avoid bias. There were baseline differences between groups but final analyses adjusted for potential confounders. | Good |
| Yotebieng et al 2015 | Y | Y | Y | N | N | Y | N | N | Y | NR | Y | Y | Y | Y | Due to nature of the interventions, blinding is not possible. Study used independent interviewers to mask data collectors and mothers to group assignments. This reportedly worked well for the mothers but not so well for the interviewers. | Good |

*Y – Yes, N – No, CD – Cannot determine/ cannot tell, NR – not reported, NA – not applicable*

1. Was the study described as randomized, a randomized trial, a randomized clinical trial, or an RCT?
2. Was the method of randomization adequate (i.e., use of randomly generated assignment)?
3. Was the treatment allocation concealed (so that assignments could not be predicted)?
4. Were study participants and providers blinded to treatment group assignment?
5. Were the people assessing the outcomes blinded to the participants' group assignments?
6. Were the groups similar at baseline on important characteristics that could affect outcomes (e.g., demographics, risk factors, co-morbid conditions)?
7. Was the overall drop-out rate from the study at endpoint 20% or lower of the number allocated to treatment?
8. Was the differential drop-out rate (between treatment groups) at endpoint 15 percentage points or lower?
9. Was there high adherence to the intervention protocols for each treatment group?
10. Were other interventions avoided or similar in the groups (e.g., similar background treatments)?
11. Were outcomes assessed using valid and reliable measures, implemented consistently across all study participants?
12. Did the authors report that the sample size was sufficiently large to be able to detect a difference in the main outcome between groups with at least 80% power?
13. Were outcomes reported or subgroups analyzed prespecified (i.e., identified before analyses were conducted)?
14. Were all randomized participants analyzed in the group to which they were originally assigned, i.e., did they use an intention-to-treat analysis?
