# Supplementary material for: Barriers and facilitators for early and exclusive breastfeeding in health facilities in Sub-Saharan Africa: a systematic review
Source: Glob Health Res Policy. 2021 Jul 6;6:21. doi: 10.1186/s41256-021-00206-2 (PMC8259208; doi:10.1186/s41256-021-00206-2)
Supplement: Supplementary file 6 — Additional file 6 : Table S6. Barriers and facilitators to facility-based breastfeeding support in Sub-Saharan Africa reported in each study. [file 41256_2021_206_MOESM6_ESM.docx]

**Table S6: Barriers and facilitators to facility-based breastfeeding support in sub-Saharan Africa reported in each study**

|  | **Barriers** | **Facilitators** |
| --- | --- | --- |
| Agbozo et al 2019 | HEALTH FACILITIES INFASTRUCTURE AND SUPPLIES   - Overcrowding and lack of space – Surge in caesarean births, overcrowding and insufficient equipment encouraged staff to quickly move mothers out of delivery to make room for the next   POLICIES AND THEIR IMPLEMENTATION   - Poor leadership and management structures - Non-autonomy of hospitals inhibited proactive implementation; Management changes compounded by poor succession plans and poor recordkeeping - Inability to sustain skilled staff with due to staffing and training policies - Was hard to achieve the recommended 20 hr training due to high staff turnover, role transfers, and funding constraints   HEALTH WORKER ENGAGEMENT   - Gaps in knowledge, misconceptions and inconsistent messaging – *Formula:* Staff did not understand rationale for why gifts from formula companies should be avoided;  *HIV+ mothers:* Gaps in knowledge on key breastfeeding issues to discuss with HIV+ mothers | POLICIES AND THEIR IMPLEMENTATION   - Commitment and leadership - Strong political commitment exemplified by the Legislative Instrument on Breastfeeding Promotion - Clear and consistent guidelines with adequate dissemination - Availability of a National Breastfeeding Policy - Mechanisms of regulation and supervision - Strict regulation through the National Breastfeeding Authority - Policy implementation-   *Rooming in*: Practice of rooming in policies was observed to be fully met in the evaluation |
| Aghaji 2002 | CAREGIVER ENGAGEMENT   - Misconceptions, beliefs and cultural practices – *Giving water:* Perceptions of giving water as common practice - Insufficient milk production - Perceptions that mother is producing insufficient milk to support infant - Peer pressure by relatives and lack of mother decision-making power – Influence of grandmother/ father | CAREGIVER ENGAGEMENT   - Maternal characteristics - *Parity:* Mother of lower parity *Education:* higher education (secondary or tertiary level) |
| Akuse and Obinya 2002 | HEALTH WORKER ENGAGEMENT   - Gaps in knowledge, misconceptions and inconsistent messaging –  *Pre-lacteal feeds:* **Non-medical staff in particular** gave pre-lacteal feed for non-medical reasons such as so the mother could rest or to quench thirst of newborn   CAREGIVER ENGAGEMENT   - Insufficient milk production – Perceived milk insufficiency | HEALTH WORKER ENGAGEMENT   - Positive attitude and willingness for breastfeeding support **-** Positive attitudes towards breastfeeding and counselling mothers |
| Amadhila and Rensburg 2020 | HEALTH FACILITIES INFASTRUCTURE AND SUPPLIES   - Overcrowding and lack of space – “Sometimes you find that there are a lot of deliveries, there is no space and you are forced to discharge some mothers, to make space.”   POLICIES AND THEIR IMPLEMENTATION   - Inability to sustain skilled staff with due to staffing and training policies – One of the main reasons reported for partial implementation were inadequate capacity in terms of training.   HEALTH WORKER ENGAGEMENT   - Staffing shortages and workload – Short staffing led to challenges to deliver appropriate breastfeeding support “With staff shortage, you cannot really pay attention, especially to the primigravidas, to show them how to properly put babies on breast.” - Gaps in knowledge, misconceptions and inconsistent messaging – *HIV+ mothers:* The huge shift in the recommendations on feeding babies of HIV infected women, from replacement feeding in 2005 to full breastfeeding in 2011, has created uncertainty and doubt among health workers   CAREGIVER ENGAGEMENT   - Fear of HIV transmission or stigma –  *HIV transmission:* “Most mothers are so worried, they want to breastfeed, but on the other hand they are afraid for the baby to get the virus.” | POLICIES AND THEIR IMPLEMENTATION   - Adequate training and staffing policies and allocation - *BHFI training:* Participants suggested that in order to strengthen the programme, there should be continuous professional development |
| Amsalu et al 2019 | HEALTH FACILITIES INFASTRUCTURE AND SUPPLIES   - Overcrowding and lack of space – Lack of space in maternity ward led to shorter stays and less time for HCWs to provide education   POLICIES AND THEIR IMPLEMENTATION   - Inability to sustain skilled staff with due to staffing and training policies - Skilled birth attendant was not regularly available to support initiation after birth (only 18.7%)   CAREGIVER ENGAGEMENT   - Gaps in knowledge due to lack of counselling –  *Lack of postpartum counselling:* Lack of health education on breastfeeding provided prior to discharge | CAREGIVER ENGAGEMENT   - Acceptability and knowledge – *Positive attitudes and familiarity:* Breastfeeding was the most recalled newborn care method by mothers (62.6%) |
| Awi and Alikor 2006 | HEALTH WORKER ENGAGEMENT   - Gaps in knowledge, misconceptions and inconsistent messaging – *Caesarean section:* Misconception that caesarean section mothers are "too ill" to early initiate   CAREGIVER ENGAGEMENT   - Health conditions of mother/infant –  *Caesarean section:* Caesarean delivery associated with delayed contact between mother and baby | POLICIES AND THEIR IMPLEMENTATION   - Adequate training and staffing policies and allocation -  *Increasing number of skilled staff:* Increased number of delivery attendants   CAREGIVER ENGAGEMENT   - Received postpartum health worker counselling and/or support - Assistance of early initiation within 30min of delivery |
| Chabeda et al 2020 | HEALTH FACILITIES INFASTRUCTURE AND SUPPLIES   - Lack of privacy or quiet place to breastfeed - Lack of conducive space within their hospital for assisting mothers with breastfeeding, especially lack of a private space to practically show mothers how to express milk and offer back rubs as a way of stimulating an oxytocin reflex in mothers who struggled with milk production.   HEALTH WORKER ENGAGEMENT   - Staffing shortages and workload – insufficient staff to allow dedicated time to support mothers to re-establish exclusive breastfeeding | POLICIES AND THEIR IMPLEMENTATION   - Adequate training and staffing policies and allocation –  *Task-shifting:* Use of breastfeeding peer supporters provided additional support for relactation, was a visible reminder of the importance of nutritional rehabilitation, were from the local community and able to speak to mothers in a language they understood, social position created trust with mothers, likely to have experienced same issues themselves |
| Chale et al 2016 | POLICIES AND THEIR IMPLEMENTATION   - Lack of guidelines/policies or their limited implementation - Limited or no availability of exclusive breastfeeding protocol; Health workers were not aware of national breastfeeding policy   HEALTH WORKER ENGAGEMENT   - Gaps in knowledge, misconceptions and inconsistent messaging –  *General:* Gaps in breastfeeding knowledge among health workers | POLICIES AND THEIR IMPLEMENTATION   - Adequate training and staffing policies and allocation –  *Hands-on training:* On- job training |
| Chaponda et al 2017 | HEALTH WORKER ENGAGEMENT   - Gaps in knowledge, misconceptions and inconsistent messaging – *HIV+ mothers:* Inconsistent knowledge from nurses regarding breastfeeding practices for HIV mothers and providing formula for HIV+ mothers. - Poor respectful maternity care– nurses are considered dominant in the postnatal ward setting, mothers were ‘told’ what to do and were shouted at when they were not conforming   CAREGIVER ENGAGEMENT   - Misconceptions, beliefs and cultural practices – *Formula:* Mothers believe that formula is "healthier - Insufficient milk production - Think that breast milk quantity is insufficient to satisfy child - Peer pressure by relatives and lack of mother decision-making power – Mothers lived mostly with their relatives. Relatives, especially grandmothers, strongly influenced feeding method. Mother felt like they had no choice but to adopt their proposed method. | CAREGIVER ENGAGEMENT   - Supportive social networks and peer support groups -  *HIV+ peers:* Seeing other HIV+ relatives breastfeed their children supported their decision to breastfeed |
| Daniels and Jackson 2011 | POLICIES AND THEIR IMPLEMENTATION   - Poor leadership and management structures - Managers felt it was extra work to implement BFHI - Inability to sustain skilled staff with due to staffing and training policies - Not enough ongoing training (workshops, visual aids, etc)   HEALTH WORKER ENGAGEMENT   - Staffing shortages and workload – Short staffing led to challenges to deliver appropriate breastfeeding support - Gaps in practical skills and management of complications – Majority of nursing staff were not able to adequately demonstrate correct hand milk-expressing technique, report about the correct management of painful nipples or how to manage breast engorgement - Poor health worker attitude or willingness – Significant number of nurses felt that implementing BFHI was an extra burden, causes “more work/energy” and managers reported that nursing resistance was a main barrier to implementation | POLICIES AND THEIR IMPLEMENTATION   - Adequate training and staffing policies and allocation - *BHFI training:* BHFI training was associated with statistically significant high rate of knowledge on feeding options for HIV+ mothers, showing how to express milk and management of painful nipples - Commitment and leadership - 87.5% believed that support from management had a positive effect on the implementation of the BFHI |
| Degefa et al 2019 | CAREGIVER ENGAGEMENT   - Health conditions of mother/infant –  *Preterm and low birthweight infants:* 50% of preterm babies and 64% of low birthweight babies had poor attachment to mothers breast in comparison to 29% of full term babies - Maternal characteristics –   *Parity:* Primipara mothers had poorer positioning and attachment compared to multipara mothers;  *Education:* Lack of formal education among mothers was associated with poorer positioning | CAREGIVER ENGAGEMENT   - Acceptability and knowledge- *Previous knowledge from antenatal care:* 74% attended antenatal care and 68% reported previous knowledge on breastfeeding |
| Doherty et al 2019 | HEALTH WORKER ENGAGEMENT   - Poor respectful maternity care– Shouting at or insulting mothers who were struggling to initiate breastfeeding “She yelled at me, she even came to me and pulled my nipple telling me that I'm failing to breastfeed the baby. She told me to put my breast in baby's mouth. I would put it. I would say, there is nothing coming out. She said, there is no such thing.” / “They never helped me. They called me isigqala. I'm like that cow called isigqala which means I do not have milk.” - Poor health worker attitude or willingness – Inconsistent support and counselling depending on health workers on shift and their attitudes towards breastfeeding. Some where supportive while others reported incorrect or absent advice.   CAREGIVER ENGAGEMENT   - Health conditions of mother/infant –  *Breast complications:* painful breasts - Peer pressure by relatives and lack of mother decision-making power – Pressure from family members, especially the infants' grandmothers, to introduce other fluids and foods - Insufficient milk production – Perceived milk insufficiency | HEALTH WORKER ENGAGEMENT   - Providing demonstrations and follow-up on breastfeeding practice - Checking and encouraging position & attachment   CAREGIVER ENGAGEMENT   - Acceptability and knowledge- *Previous knowledge from antenatal care:* Mothers described receiving breastfeeding information during antenatal care, with a focus on exclusive breastfeeding for 6 months as main message - Received postpartum health worker counselling and/or support - Support available immediately postpartum |
| Dubik et al 2021 | POLICIES AND THEIR IMPLEMENTATION   - Inability to sustain skilled staff with due to staffing and training policies –Most (64.4%) of the nurses and midwives reported in-service training experience as their primary source of breastfeeding knowledge and a vast majority (79.8%) think they need further updating/training on breastfeeding - Lack of guidelines/policies or their limited implementation – 75% reported that lack of materials for breastfeeding was a barrier to breastfeeding counselling in their facilities   HEALTH WORKER ENGAGEMENT   - Staffing shortages and workload - 84% cited too much workload and 77% cited not enough time as key barriers to breastfeeding counselling at their facilities - Gaps in practical skills and management of complications – 39.4% had training in the management of breast conditions and other breastfeeding difficulties | POLICIES AND THEIR IMPLEMENTATION   - Adequate training and staffing policies and allocation –  *Hands-on training:* On- job training correlated positively with nurses and midwives confidence levels in counselling mothers about infant and young child feeding (*r* = .299, *P* = .002)   *Pre-service training:* Pre-service training correlated positively with nurses and midwives confidence levels in counselling mothers about infant and young child feeding (*r* = .359, *P* < .001).  HEALTH WORKER ENGAGEMENT   - Positive attitude and willingness for breastfeeding support **-** Positive attitudes towards breastfeeding and counselling mothers, nearly all felt that counselling mothers about breastfeeding was one of their job responsibilities. |
| Fadupin et al 2020 | CAREGIVER ENGAGEMENT   - Health complications –  *Illness of mother:*  complications experienced by the mothers during or before delivery significantly lowered the proportion of mothers who had early breastfeeding initiation (p꞊0.001). | POLICIES AND THEIR IMPLEMENTATION   - Policy implementation-   *Rooming in*: Policy implementation of having mothers roomed with the child one hour after delivery (p꞊0.022) significantly associated with the breastfeeding initiation  CAREGIVER ENGAGEMENT   - Received postpartum health worker counselling and/or support – receiving education on early initiation of breastfeeding (p꞊0.025) and help from the healthcare providers on initiation (p꞊0.000) significantly associated with breastfeeding initiation |
| Ferguson et al 2009 | POLICIES AND THEIR IMPLEMENTATION   - Inability to sustain skilled staff with due to staffing and training policies – Inadequate training and staffing policies with high patient-to-nurse ratio   HEALTH WORKER ENGAGEMENT   - Staffing shortages and workload – nurses reported struggling to provide adequate counselling due to high workload. There were typically two nurses for an average of 31 mothers and 16 infants waiting for counselling and other health services | POLICIES AND THEIR IMPLEMENTATION   - Adequate training and staffing policies and allocation –  *Increasing numbers of skilled staff:* Recommendation for more nurses in the clinic - Mechanisms of regulation and supportive supervision - Having a supervisor or evaluator monitor and observe their counseling sessions was suggested by nurses to support improving implementation adherence.   HEALTH WORKER ENGAGEMENT   - Positive attitude and willingness for breastfeeding support **-** Nurses reported that counselling mothers on breastfeeding was “easy” |
| Gejo et al 2019 | CAREGIVER ENGAGEMENT   - Health complications –  *Breast complications:* breast engorgement *Illness of mother or infant:*  Illness of mother or child: - Insufficient milk production – Perceived milk insufficiency - Fear of HIV transmission or stigma –  *HIV transmission:* reported as the top reason given for not breastfeeding among this group of HIV+ women | CAREGIVER ENGAGEMENT   - Acceptability and knowledge-  P*revious knowledge from antenatal care:* Infant feeding counselling during ANC and had knowledge of exclusive breastfeeding practices *Positive attitudes and familiarity: p*ositive attitude & knowledge of EBF practices |
| Getnet et al 2020 | CAREGIVER ENGAGEMENT   - Health conditions of mother –  *Caesarean section:* Almost half of caesarean mothers in the study (48.1%) did not initiate breastfeeding in the first hour, with 58% of those who did not initiate breastfeeding early citing caesarean surgery-related pain and discomfort. - Insufficient milk production – Delayed milk secretion cited by caesarean mothers as a main reason for not initiating breastfeeding early | CAREGIVER ENGAGEMENT   - Acceptability and knowledge –   *Positive attitudes and familiarity:* Caesarean mothers with previous breastfeeding experience were more likely to initiate breastfeeding early (AOR = 2.25, 95% CI = 1.33, 3.75)  *Previous knowledge from antenatal care:* Caesarean mothers who had four and above visits were more likely to initiate breastfeeding early as compared to those mothers who had less than four visits (AOR = 2.20, 95% CI = 1.24, 3.91)   - Received postpartum health worker counselling and/or support– Caesarean mothers who had received professional guidance were more likely to initiate breastfeeding early (AOR= 2.68, 95% CI = 1.18, 6.10) |
| Hasselberg et al 2016 | POLICIES AND THEIR IMPLEMENTATION   - Inability to sustain skilled staff with due to staffing and training policies – Premature infant ward is unstaffed at night   CAREGIVER ENGAGEMENT   - Health conditions of mother/infant –  *Preterm and low birthweight infants:* Difficulties with preterm infants “I am used to breastfeeding, but not this small baby.” – difficulties suckling/ coughing, aspiration, or apnea episodes after feeding/ fear about holding small newborn; *Caesarean section:*  Pain after caesarean delivery, maternal complications that led to longer stays in maternity; *Breast complications:* Nipple pain and breast encouragement especially with expressed breastmilk | HEALTH FACILITIES INFASTRUCTURE AND SUPPLIES   - Supplies that support breastfeeding practice – wall clock and cell phone alarm to support feeding infants every two hours   HEALTH WORKER ENGAGEMENT   - Providing respectful maternal care – health workers providing empathetic and compassionate care - Providing demonstrations and follow-up on breastfeeding practice - The mothers said that the doctor sometimes corrected them, for instance, if they saw that the infants did not suck the right way   CAREGIVER ENGAGEMENT   - Learning skills and techniques to improve breastfeeding practice –   *Expressed breastmilk* - Counselling on how to increase expression of milk *Timed strategies* – support to feed infants every two hours and teaching women to read clocks to support their own practice  *To enhance nutrition –* teaching good nutrition to mothers and their families to support breastfeeding   - Supportive social networks and peer support groups -  *Peer:* Support from other mothers in the hospital preterm ward *Family:* Support from family, bringing food, female family members supporting care in ward |
| Ighogboja et al 1996 | CAREGIVER ENGAGEMENT   - Gaps in knowledge due to lack of counselling –  *Lack of or inadequate postpartum counselling:* Postpartum breastfeeding support and demonstrations were rarely given;  *Lack of or inadequate counselling in antenatal care:* Many who attended private clinics had no breastfeeding education. - Insufficient milk production – 51% of those who delayed breastfeeding past 24 hours reported that it was due to failure to produce breastmilk - Health conditions of mother/infant –  *Caesarean section:*  24% of those who delayed breastfeeding past 24 hours was due to operative surgery - Misconceptions, beliefs and cultural practices – *Formula:* A fifth of mothers (21%) discarded colostrum | CAREGIVER ENGAGEMENT   - Acceptability and knowledge-  P*revious knowledge from antenatal care:* caregivers had higher levels of BF knowledge after attending antenatal care - Received postpartum health worker counselling and/or support - Facility births had increased opportunities for counselling, which was associated with higher rates of breastfeeding |
| Iliyasu et al 2019 | CAREGIVER ENGAGEMENT   - Fear of HIV transmission or stigma –  *HIV transmission:* major gaps in maternal knowledge and infant feeding practices for HIV+ mothers | CAREGIVER ENGAGEMENT   - Received postpartum health worker counselling and/or support - Facility births had increased opportunities for counselling and was associated 3x odds of exclusive breastfeeding |
| Kafulafula et al 2014 | CAREGIVER ENGAGEMENT   - Fear of HIV transmission or stigma –  *HIV transmission:* 15 of 16 HIV+ mothers expressed fear of transmitting HIV to their infants, of having frequently sick infants or death of their infants if they exclusively breastfed them - Misconceptions, beliefs and cultural practices – *Harms:* Perceptions that exclusive breastfeeding is very demanding on the mothers’ body with potential of weakening their health ad making them develop AIDs faster | CAREGIVER ENGAGEMENT   - Acceptability and knowledge-  *Positive attitudes and familiarity:* positive perception on how maternal health affected by EBF (better nutritional status, self-satisfying, form of birth control), positive perception on how baby health affected by EBF (promotes well-being, love/connection to mom, prevent MTCT of HIV) |
| Kahindi et al 2020 | CAREGIVER ENGAGEMENT   - Health conditions of mother – *Maternal emotional stress:* some mothers found the ward environment stressful, especially those with previous negative ward experiences such as the death of a child | POLICIES AND THEIR IMPLEMENTATION   - Adequate training and staffing policies and allocation –  *Task-shifting:* Use of breastfeeding peer supporters seen by mothers as more approachable than other health workers and easier to communicate with   CAREGIVER ENGAGEMENT   - Learning skills and techniques to improve breastfeeding practice –   *Expressed breastmilk* - Counselling on how to increase expression of milk   - Received postpartum health worker counselling and/or support – particularly from breastfeeding peer support workers |
| Kalisa et al 2015 | CAREGIVER ENGAGEMENT   - Gaps in knowledge due to lack of counselling –  *Lack of or inadequate counselling in antenatal care:* Inadequate prenatal guidance associated with delayed initiation (aOR 3.6, 95% CI: 1.9-6.8) - inadequate information on early initiation given to mothers during ANC visits, which focused on exclusive breastfeeding, PMTCT and encouraging mothers to deliver at health facilities - Health conditions of mother/infant –  *Caesarean section:* Delayed initiation of breastfeeding associated with caesarean section delivery (aOR 8.6, 95% CI: 4.7-16.0) because of being exhausted, sleepy and severe abdominal pain from the wound. Caesarean section babies given pre-lacteal feeds (sugar-water solution) until mothers’ pain subsides. “I started to breast feed after the abdominal pain, dizziness and weakness had reduced and l could sit to breastfeed the baby...” - Insufficient milk production – Lack of or insufficient breast milk reported by most mothers “…it would be unfair to give the baby an empty breast to satisfy the infant...” - Difficulty with breastfeeding practice and receiving inadequate health worker support - Inadequate professional assistance to initiate breastfeeding associated with delayed breastfeeding (aOR 1.8, 95% CI: 1.2-2.8) - Misconceptions, beliefs and cultural practices –  *Colostrum*: Colostrum as dirty, needed to wait until second day until “first breast milk mixes with the second breast milk”   *Rest:* the baby needs to rest after delivery   - Peer pressure by relatives and lack of mother decision-making power – Influence of infant’s grandmothers “because I had been told by my mother to wait” - Fear of HIV transmission or stigma –  *HIV transmission:* maternal HIV positive status (AOR 2.3; 95% CI 1.3-4.2) “…before delivery I had decided to breastfeed immediately as we had been taught during ANC but after seeing my baby I delayed to decide on whether to start breast feeding or not….” | POLICIES AND THEIR IMPLEMENTATION   - Commitment and leadership - Governmental/non-government initiatives to promote EIBF |
| Kassa et al 2021 | CAREGIVER ENGAGEMENT   - Insufficient milk production – Delayed milk secretion cited by a third of mothers with late initiation | CAREGIVER ENGAGEMENT   - Acceptability and knowledge –   *Positive attitudes and familiarity:* Mothers with previous breastfeeding experience were more likely to initiate breastfeeding within one hour than those who did not (AOR=1.79, 95% CI=1.19, 2.68)   - Received postpartum health worker counselling and/or support– Mothers who had received professional guidance were more likely to initiate breastfeeding early as compared to those who did not receive support (AOR=2.75, 95% CI=1.20, 6.34) |
| Kavle et al 2019 | HEALTH FACILITIES INFASTRUCTURE AND SUPPLIES   - Overcrowding and lack of space – Overcrowding due to high patient volume led to decreased counselling   POLICIES AND THEIR IMPLEMENTATION   - Inability to sustain skilled staff with due to staffing and training policies - Lack of BFHI funding led to lack of training and staffing. BFHI initiative deteriorated following loss of funding in 2004, which was associated with decreased rate of exclusive breastfeeding rate 2010 (71%) to 2015/2016 (61%). Frequent staff transfers to different facilities and staff member often replaced with someone not trained. Lack of proper training prevented adequate support and training for early and exclusive breastfeeding. In particular, **nonclinical support staff** were not trained. - Lack of guidelines/policies or their limited implementation - Many hospitals have not translated the policy into languages commonly spoken within the catchment area   HEALTH WORKER ENGAGEMENT   - Staffing shortages and workload - Under‐resourced health facilities and staff overburdened with high patient load   CAREGIVER ENGAGEMENT   - Gaps in knowledge due to lack of counselling –  *Lack of or inadequate counselling in antenatal care:* Starting ANC late is common, which decreased exposure to BFHI messages - Health conditions of mother/infant –  *Caesarean section:* Complications associated with caesarean births led to reduced skin‐to‐skin care - Insufficient milk production – Perceptions of insufficient breastmilk, leading babies to cry out from hunger - Difficulty with breastfeeding practice and receiving inadequate health worker support - Poor positioning and attachment - Misconceptions, beliefs and cultural practices –  *Giving water*: Giving water because "baby is thirsty", especially during warmer months *Cultural traditions:* Cultural tradition of giving pre-lacteal feeds (phala ufawoyera watery refined porridge) | POLICIES AND THEIR IMPLEMENTATION   - Commitment and leadership - Community leadership, created a task force at each hospital - Mechanisms of regulation and supportive supervision - weekly planning, monitoring and implementation meetings - Adequate training and staffing policies and allocation –   *Hands-on training:* Increasing number of "master" trainers to teach health workers at the district level  *Task-*shifting*:* Encouraged task shifting of breastfeeding counselling and promotion to support staff to overcome challenges of short staffing. Over 600 support staff from 18  facilities were trained on BFHI. |
| Kusi-Amponsah Diji et al 2017 | CAREGIVER ENGAGEMENT   - Health conditions of mother/infant –  *Breast complications:* Cracked or sore nipples, breast engorgement *Maternal emotional stress*: Maternal emotional stress - Insufficient milk production – Perceptions of low breastmilk production - Difficulty with breastfeeding practice and receiving inadequate health worker support – Lack of support from healthcare professionals - Misconceptions, beliefs and cultural practices –  *Insufficient nutrition*: Perception that breastmilk alone would not meet nutritional needs for baby *Cultural traditions:* Socio-cultural pressure to introduce pre-lacteal feeds |  |
| Lang'at et al 2018 | HEALTH WORKER ENGAGEMENT   - Gaps in knowledge, misconceptions and inconsistent messaging – *HIV+ mothers:* Healthcare workers from the same health facility had divergent information on the current guideline recommendations with some still using old guidelines. Some reported that modified cow’s milk could be used as a replacement. - Poor health worker attitude or willingness – Feeding issues perceived to be duties of the nutritionist. Clinicians, nurses and adherence counsellors referred pregnant HIV+ mothers to the nutritionist for teaching   CAREGIVER ENGAGEMENT   - Misconceptions, beliefs and cultural practices –  *Cultural traditions*: ‘When we give birth, the first thing that we give our babies is “Kerichek ap kip kaa” (traditional herbs used by the Kalenjin community in Kenya) to help flush out dirt that has accumulated in the baby’s stomach while in the womb’. - Peer pressure by relatives and lack of mother decision-making power – Social pressure from the family members as the main reason for the administration of “Kerichek ap kip kaa” - Fear of HIV transmission or stigma –  *Stigma:* Non-disclosure of HIV in the community was a barrier to community support | NEGATIVE FACILITATOR   - Economic constraints –Some HIV+ mothers reported that they exclusively breastfeed because they could not afford infant formula and/or limited access to safe water for its preparation |
| Mgolozeli et al 2019 | HEALTH WORKER ENGAGEMENT   - Gaps in knowledge, misconceptions and inconsistent messaging – *Potential harms:* Some thought that skin-to-skin contact causes hypothermia - Poor health worker attitude or willingness – Almost half of nursing staff (45%) thought that exclusive breastfeeding was for poor people; **enrolled nursing auxiliaries** in particular had negative attitudes towards mother-baby friendly hospital initiatives and the promotion of breastfeeding (thought it was time consuming, not their responsibility, did not believe in or teach women about benefits) | HEALTH WORKER ENGAGEMENT   - Positive attitude and willingness for breastfeeding support - Majority of nurses (52%) had positive attitudes (professional & enrolled nurses mainly) and was strongly against the use of pre-lacteal feeds including water |
| Mohamed et al 2018 |  | HEALTH WORKER ENGAGEMENT   - Positive attitude and willingness for breastfeeding support - Positive attitude of exclusive breastfeeding - Good knowledge about breastfeeding benefits and practices -  *General:* high level of knowledge of exclusive breastfeeding practices |
| Morgan and Jeggels 2015 | POLICIES AND THEIR IMPLEMENTATION   - Lack of guidelines/policies or their limited implementation - Free state-provided formula delivers a contradicting message to HIV-positive mothers and its phasing out was not clearly explained to health workers. Insufficient coordination between PMTCT programme policies and local practices and grassroots level health workers may not be aware of the latest policy changes   HEALTH WORKER ENGAGEMENT   - Gaps in knowledge, misconceptions and inconsistent messaging – *HIV+ mothers:* Inconsistent messaging on infant feeding practices for HIV-positive mothers   CAREGIVER ENGAGEMENT   - Gaps in knowledge due to lack of counselling –  *Lack of or inadequate postpartum counselling:* Postpartum infant feeding counselling was not received by a majority of women (62%) | POLICIES AND THEIR IMPLEMENTATION   - Policy implementation –   *Formula and/or mixed feeding:* Removal of free formula to HIV+ mothers after updated policies  CAREGIVER ENGAGEMENT   - Acceptability and knowledge-  *Receptiveness to health worker counselling:* women considered health workers as the primary source of feeding info |
| Morhason-Bello et al 2009 | POLICIES AND THEIR IMPLEMENTATION   - Inability to sustain skilled staff with due to staffing and training policies - Not enough skilled birth attendants   HEALTH WORKER ENGAGEMENT   - Staffing shortages and workload - reported that staffing shortages was associated with women not getting the necessary attention to facilitate early initiation | CAREGIVER ENGAGEMENT   - Received postpartum health worker counselling and/or support - Assisting women in pain after birth for proper attachment and positioning - Supportive social networks and peer support groups -  *Family:* First time mothers with birth companions was associated with significantly earlier time to breastfeeding initiation compared to controls without birth companions (p < 0.01). |
| Moussa et al 2010 | HEALTH FACILITIES INFASTRUCTURE AND SUPPLIES   - Overcrowding and lack of space – Consulting room did not have dedicate space for feeding - Lack of privacy or quiet place to breastfeed - Unprofessional environment causing distraction during consultation (eg. Auction sales) - Insufficient equipment or supplies - No chairs, changing tables, poor facility infrastructure and only one guard with access to water tank during water cut-offs. No back up generator for power cuts.   POLICIES AND THEIR IMPLEMENTATION   - Inability to sustain skilled staff with due to staffing and training policies - Poor training coverage and staffing shortages led to often recruiting volunteers and trainees to assist with deliveries. - Lack of guidelines/policies or their limited implementation - Adherence to breastfeeding protocols not enforced. Some health facilities had posters promoting formula and delegates from pharmaceutical companies promoted misleading info of the benefits of formula to health workers   HEALTH WORKER ENGAGEMENT   - Staffing shortages and workload – short staff and inadequate time to adequately support breastfeeding - Health worker attitudes and willingness - Prioritize other tasks over BF counselling. Hastily done advice/consultation sessions with no direct mentioning of exclusive breastfeeding benefits. Some health providers do not believe in exclusive breastfeeding and encourage the use of breast milk subsitutes. - Gaps in knowledge, misconceptions and inconsistent messaging –  *Pre-lacteal feeds:* Promoting mixed feeding to some mothers, belief that giving water for heat is okay   CAREGIVER ENGAGEMENT   - Difficulty with breastfeeding practice and receiving inadequate health worker support No assistance to initiate BF in first half hour after birth and did not provide advice on positioning - Misconceptions, beliefs and cultural practices - *Giving water*: belief that water is necessary during hot season | POLICIES AND THEIR IMPLEMENTATION   - Policy implementation –   *Skin-to-skin:* promoted immediate skin to skin contact after labour *Formula and/or mixed feeding:* Took away any equipment which promoted mixed feeding   - Adequate training and staffing policies and allocation –   *Task-shifting:* Femme relais (FR) are volunteers who are generally older, trained by health professionals and have experience with childbirth - they were often the ones providing advice on BF |
| Mphasha and Skaal 2019 | POLICIES AND THEIR IMPLEMENTATION   - Inability to sustain skilled staff with due to staffing and training policies – Majority of nurses (64%) not trained on updated guidelines - Lack of guidelines/policies or their limited implementation - An overwhelming majority of nurses reported that clinics still stored and issued infant formula to HIV-positive mothers despite clear changes in the revised IYCF policies to not do so. 45% reported that clinical did not have a copy of the HIV/IYCF guideline and 20% were not sure   HEALTH WORKER ENGAGEMENT   - Health worker attitudes and willingness – A significant portion of respondents reported good knowledge but poor practices - Gaps in knowledge, misconceptions and inconsistent messaging –  *HIV+ mothers:* Most nurses have not been trained on updated guidelines and nurses may be confused about the changes made to HIV/ IYCF recommendations   *Overall gap in knowledge:* None of the nurses with poor knowledge displayed good practices | HEALTH WORKER ENGAGEMENT   - Good knowledge about breastfeeding benefits and practices –   *Infant feeding and HIV+:* almost all participants displayed good knowledge regarding the revised HIV/IYCF policy (2013) including agreement that HIV exposed children should be exclusively breastfeed for six months, no water should be given during EBF, that formula feeding should only be recommended due to medical reasons and that breastfeeding is best for both HIV exposed and unexposed children |
| Mukerem and Haidar 2012 | CAREGIVER ENGAGEMENT   - Health conditions of mother/infant –  *Breast complications:* Dryness of their breast *Illness of mother or infant*: Mothers who experienced illness were 74% less likely to exclusively breastfeed than who had no illness (aOR 0.26, 95% CI: 0.13-0.53). - Fear of HIV transmission or stigma –   *HIV transmission*: Fear of transmission given by 80% mothers who stopped exclusive breastfeeding early   - Gaps in knowledge due to lack of counselling –  *Lack of or inadequate counselling during antenatal care:* Of the mothers who stopped breastfeeding early, only 20% received advice from health professionals - Misconceptions, beliefs and cultural practices – *Negative attitude in general:* Mothers who had negative attitudes towards exclusive breastfeeding were 68% less likely to adhere to exclusive breastfeeding than those who had positive attitudes. (AOR=0.32; 95%CI= 0.16-0.63) - Peer pressure by relatives and lack of mother decision-making power – External pressure from husbands, family and neighbors | CAREGIVER ENGAGEMENT   - Acceptability and knowledge-  *Receptiveness to health worker counselling: M*ost mothers adhered to exclusive breastfeeding practice because of the advice of health professionals - Supportive social networks and peer support groups -  *Family:* the support from their spouses and family.   *HIV+ peers:* mother support group services delivered through weekly coffee ceremony at health facility |
| Mukashyaka et al 2020 | CAREGIVER ENGAGEMENT   - Health conditions of mother –  *Caesarean section:* caesarean section reported as major reason for delayed breastfeeding as the practice of early initiation to breastfeeding was rarely practiced post-surgery in Rwanda - Insufficient milk production – Delayed milk secretion cited by 41.9% for not exclusively breastfeeding - Difficulty with breastfeeding practice and receiving inadequate health worker support – requiring and not receiving adequate help to breastfeeding, such as with positioning and latching and expressing colostrum, was a main reason for delayed breastfeeding |  |
| Nabwera et al 2017 | POLICIES AND THEIR IMPLEMENTATION   - Inability to sustain skilled staff with due to staffing and training policies – Inadequate training on updated guidelines - Lack of guidelines/policies or their limited implementation - Changing policies on HIV/ IYCF reached health workers in rural or peripheral facilities more slowly than those working in larger or more urban facilities   HEALTH WORKER ENGAGEMENT   - Staffing shortages and workloads - overwhelming workloads resulting in inadequate time for counseling and long waiting times for mothers - Gaps in knowledge, misconceptions and inconsistent messaging –  *HIV+ mothers:* Health workers frequently shared that ‘constantly changing policies and guidelines’ led to confusion and inconsistent messaging   CAREGIVER ENGAGEMENT   - Fear of HIV transmission or stigma –   *Stigma*: Mothers often lived with their extended families. In this context women living with HIV who had not disclosed their status to their family members found it difficult to undertake feeding practices that departed from the norm   - Peer pressure by relatives and lack of mother decision-making power – Mothers often lacked autonomy in the decision-making processes around infant feeding in the context of HIV. Children perceived as “belonging” to the father though fathers rarely attended infant feeding counseling sessions and had limited knowledge | CAREGIVER ENGAGEMENT   - Acceptability and knowledge-  *Previous knowledge from antenatal care:* Contact with the health facilities especially during antenatal care visits was reported by carers to have been highly influential in their infant feeding decision-making. - Supportive social networks and peer support groups -  *HIV+ peers:* HIV+ mothers expressed the value that they derived from a facility initiated peer group that helped to mitigate the challenges that they experienced with adhering to the infant feeding guidance. Support from peers also provided mothers with more information about the infant feeding recommendations, apparently influencing infant feeding choices. Women living with HIV enrolled in a mother-to-mother (M2M) support group reported being better able to adhere to feeding recommendations. |
| Nii Okai Aryeetey and Antwi 2013 | POLICIES AND THEIR IMPLEMENTATION   - Poor leadership and management structures - Lack of strong leadership and sustained program planning. Monitoring often left unattended. - Inability to sustain skilled staff with due to staffing and training policies - Lack of funds was given as the reason for gaps in training to build staff capacity. There were only a few trained national assessors to go round the entire nation to perform supportive monitoring as well as assessments and training. Problems of high trained staff attrition and inadequate in-service training for new staff - Lack of guidelines/policies or their limited implementation - Poor adherence of the facilities to the Ten Steps to successful breastfeeding. Though a written BHFI policy existed, gap in routinely communicating policy to staff or displayed at relevant places for the benefit of staff and clients   HEALTH WORKER ENGAGEMENT   - Staffing shortages and workload – high client-staff ratios lead to heavy workloads on existing staff and inadequate opportunity to support breastfeeding counselling and practice - Gaps in knowledge, misconceptions and inconsistent messaging –  *Formula:* Health staff had poor knowledge on the reasons for not permitting distribution of free formula samples - Gaps in practical skills and management of complications- Lack of training in skills necessary to implement the BHFI policy including showing mothers how to breastfeed and maintain lactation - Health worker attitudes and willingness – Staff are less motivated to participate in training that is not linked with extra income | POLICIES AND THEIR IMPLEMENTATION   - Policy implementation-   *Rooming in*: rooming-in was adhered to by all facilities, which was aided by space constraints in most hospitals that facilitated the keeping of infants with their mothers (NEGATIVE FACILITATOR) |
| Nikodem et al 1995 | POLICIES AND THEIR IMPLEMENTATION   - Inability to sustain skilled staff with due to staffing and training policies - Less than half (43%) of hospitals had staff members who have specialised training in breastfeeding and lactation management available full-time to assist mothers. - Lack of guidelines/policies or their limited implementation – Under half (47 % ) of hospitals explicit written policy for protecting, promoting and supporting breastfeeding and only about a third (35%) had a mechanism for evaluating the effectiveness of the policy. Over half of mothers reported they were not given the opportunity to put their babies to the breast for the first time within one hour after delivery   HEALTH WORKER ENGAGEMENT   - Gaps in knowledge, misconceptions and inconsistent messaging –  *Overall gap in knowledge:* Conflicting advice from health workers - Health worker attitudes and willingness – Lack of health worker support, restricting of feeding - Poor respectful maternity care - Mothers reported reduction of self-esteem by the patronising approach of health care workers | HEALTH WORKER ENGAGEMENT   - Positive attitude and willingness for breastfeeding support - 72% said they help mothers of babies in special care to establish and maintain lactation by frequent expression of milk. A majority of mothers felt that staff were supportive of breastfeeding. - Good knowledge about breastfeeding benefits and practices –   *General:* 75% of mothers who responded felt that staff were knowledgeable   - Providing demonstrations and follow-up on breastfeeding practice – staff helped to establish and maintain lactation   CAREGIVER ENGAGEMENT   - Learning skills and techniques to improve breastfeeding practice –   *Expressed breastmilk* - Counselling on how establish and maintain lactation by frequent expression of milk   - Received postpartum health worker counselling and/or support - 71% of mothers responded that they were shown how to breastfeed their infants. |
| Nyati-Jokomo et al 2019 | HEALTH FACILITIES INFASTRUCTURE AND SUPPLIES   - Overcrowding and lack of space – Shortage of space at the health facilities - Lack of privacy or quiet place to breastfeed - Health professionals failed to observe confidentiality as they would ask them questions related to their HIV statuses when the other patients were listening. The women were asked to sit in their own queue and it became obvious that they were HIV positive.   CAREGIVER ENGAGEMENT   - Fear of HIV transmission or stigma – - *HIV transmission:* Feared that the virus in their milk would infect their babies and mistrusted breastfeeding their babies. Mothers mistrusted the advice they received from the nurses on the safety of breast milk.  *Stigma*: Low self-esteem because of HIV+ status, felt that health care providers were treating them differently from the rest of the other health care seekers, precedence was given to other patients - Peer pressure by relatives and lack of mother decision-making power – Community members and significant others discouraged HIV+ mothers from breastfeeding - Misconceptions, beliefs and cultural practices –   *Harms:* All the women suggested that the beliefs in the community were that an HIV infected mother’s milk was unsafe for infant feeding. | NEGATIVE FACILITATOR   - Economic constraints - Two thirds of the mothers confessed attempting to minimize breast-feeding their babies but due to economic hardships they were forced to breastfeed when they were unable to offer alternative feeds |
| Nyawade et al 2016 | HEALTH WORKER ENGAGEMENT   - Gaps in knowledge, misconceptions and inconsistent messaging –  *HIV+ mothers:* A third of health providers reported that exclusive breastfeeding would lead to HIV transmission   CAREGIVER ENGAGEMENT   - Difficulty with breastfeeding practice and receiving inadequate health worker support – Health workers rarely observed a mother breastfeed to address difficulties and misconceptions | HEALTH WORKER ENGAGEMENT   - Positive attitude and willingness for breastfeeding support - Healthier babies was valued by health workers as an outcome of breastfeeding - Good knowledge about breastfeeding benefits and practices –   *General:* Reduced childhood illnesses and better immunity for the baby   - Positive work culture and social norms among medical staff supporting breastfeeding – health workers perceived that colleagues, immediate supervisors, managers, and breastfeeding advocates as approving of their supporting mothers to exclusively breastfeed - Providing demonstrations and follow-up on breastfeeding practice -giving information and demonstrating skill/techniques - Respectful maternity care - Asking and/or listening to a mother were reported by a few health workers as ways they supported mothers to breastfeed |
| Ojofeitimi et al 2000 |  | CAREGIVER ENGAGEMENT   - Maternal characteristics   *Education* - Higher level of education was associated with higher acceptance of exclusive breastfeeding |
| Okolo and Ogbonna 2002 | POLICIES AND THEIR IMPLEMENTATION   - Lack of guidelines/policies or their limited implementation – None of the **auxiliary health workers** were aware of the existence and duties of lactation support groups and had poor awareness of early initiation of breastfeeding policies and benefits   HEALTH WORKER ENGAGEMENT   - Gaps in knowledge, misconceptions and inconsistent messaging –  *Pre-lacteal feeds:* only 19.2% of the health workers interviewed believed that babies less than 6 months of age should *not* be given water - Gaps in practical skills and management of complications- Low proportion of health workers overall (5% each) were able to demonstrate correct positioning and attachment |  |
| Olorunfemi and Dudley 2018 | CAREGIVER ENGAGEMENT   - Fear of HIV transmission or stigma –   *HIV transmission:* Almost all of the mothers who did not exclusively breastfeed reported worry of HIV transmission through breastfeeding   - Peer pressure by relatives and lack of mother decision-making power – Experienced pressure from relatives or friends to give water, formula or solid food to their infants - Health conditions of mother/infant –  *Breast complications:* Cracked or sore nipples   *Illness of mother or infant:* Mother was ill   - Insufficient milk production - Perceived milk insufficiency | CAREGIVER ENGAGEMENT   - Acceptability and knowledge-   *Positive attitudes and familiarity:* Almost all HIV+ mothers felt strongly about their preference to exclusively breastfeed and said they would still breastfeed even if provided with a free supply of formula  *Previous knowledge from antenatal care:* Mothers who received information, education and counselling on infant feeding options early during pregnancy were more likely to exclusively breastfeed as compared to mothers who received information late in their pregnancy or after delivery (p = 0.01). Mothers’ satisfaction with the information provided during the PMTCT programme was positively associated with exclusive breastfeeding practice (p = 0.006).   - Supportive social networks and peer support groups -   *Family:* Most mothers (78%) reported that their partners preferred them to exclusively breastfeed |
| Owoaje et al 2002 |  | POLICIES AND THEIR IMPLEMENTATION   - Adequate training and staffing policies and allocation - *BHFI training:* BHFI training was associated with statistically significant higher rate of knowledge in comparison to untrained nurses   HEALTH WORKER ENGAGEMENT   - Good knowledge about breastfeeding benefits and practices -   *Infant feeding and HIV+*: BFHI trained nurses were more knowledgeable on feeding options for HIV+ mothers  *Management of complications and/or specialized care:* Higher proportions of the BFHI trained nurses were more knowledgeable about the correct management of mastitis, breast abscess, breast engorgement, painful nipples, low milk supply, and breastfeeding counsel to a mother of child with neonatal jaundice.  *General:* BFHI trained nurses were more knowledgeable about the advantages of reduction in the incidence of diarrhoea in children, its cheapness, ready availability and reduction in post partum haemorrhage   - Providing demonstrations and follow-up on breastfeeding practice - showing how to express milk - Positive attitude and willingness for breastfeeding support - Higher proportions of the BFHI trained nurses had positive attitudes for positive support practices, including against giving water to exclusively breastfed infants |
| Remmert et al 2020 | CAREGIVER ENGAGEMENT   - Fear of HIV transmission or stigma –   *HIV transmission:* The most frequently cited reason for not breastfeeding | CAREGIVER ENGAGEMENT   - Supportive social networks and peer support groups –   *HIV+ peers:* Participants who had ever breastfed had higher HIV-related support (M = 6.72, SD = 0.47, range 5.25–7) than participants who had never breastfed (M =6.32, SD = 1.14, range 2.25–7; p = 0.004). Participants who were currently EBF had higher HIV-related support (M = 6.71, SD = 0.44, range 5.5–7) compared to participants currently exclusively formula feeding (M =6.42, SD = 1.04, range 2.25–7; p = 0.02).  *Family:* Participants who had ever breastfed had higher functional social support (M = 38.4, SD = 3.03, range 27–40) compared to participants who had never breastfed (M =36.55, SD = 6.32, range 10–40; p = 0.02). Rates of HIV disclosure did not differ between infant feeding practices; most women had disclosed their status to an immediate family member and/or current partner |
| Senbanjo et al 2014 | POLICIES AND THEIR IMPLEMENTATION   - Lack of guidelines/policies or their limited implementation – The hospital has a written hospital policy on breastfeeding practices, but none were posted on the wall and only half of the staff were aware that the hospital has a written policy that supports breastfeeding. There was no existing breastfeeding support group for mothers.   HEALTH WORKER ENGAGEMENT   - Poor health worker attitude or willingness – Only a quarter of staff (24%) had supported assisting mothers on how to breastfeeding in the last three months | CAREGIVER ENGAGEMENT   - Maternal characteristics –   *Religion:* Christian background (OR 3.0; 95% CI 1.7, 5.2; p < 0.001) was significantly associated with exclusive breastfeeding  *Private hospital attendance and/or socio-economic status:* Antenatal care in private hospitals (OR 3.8; 95% CI 1.2, 11.5; p = 0.029) was significantly associated with exclusive breastfeeding |
| Senghore et al 2018 | CAREGIVER ENGAGEMENT   - Maternal characteristics –   *Maternal age:* The age of the mother was significantly associated with intention to or practice of exclusive breastfeeding. Mothers aged between 26 and 34 years were found to have about twice decreased likelihood to intend to or practice EBF compared to those age ≤ 25 years | CAREGIVER ENGAGEMENT   - Acceptability and knowledge-   *Positive attitudes and familiarity:* Having a positive attitude towards exclusive breastfeeding (aOR 2.40; 95% Cl 1.40, 4.10; p = 0.003) significantly associated with increased odds of having sufficient knowledge on exclusive breastfeeding, which was associated with intention (antenatal participants) or practice (postnatal participants) of exclusively breastfeed.   - Supportive social networks and peer support groups –   *Family:* Having a partner supporting exclusive breastfeeding (aOR 2.18; 95% Cl 1.23, 3.87; p = 0.008) significantly associated with increased odds of having sufficient knowledge on exclusive breastfeeding, which was associated with intention (antenatal participants) or practice (postnatal participants) of exclusively breastfeed. Almost half reported that family members as their major source of knowledge on exclusive breastfeeding.   - Maternal characteristics –   *Private hospital attendance and/or socio-economic status:* Earning 1500 GMD or more monthly (aOR 1.98; 95% Cl 1.24, 3.16; p = 0.004)  ** Counseling on EBF (aOR 2.68; 95% Cl 1.68, 4.29; p < 0.001) was also significantly associated with intention to or practice of exclusive breastfeeding 🡪 but not described if this was antenatal or postnatal counselling or both. |
| Spira et al 2017 |  | POLICIES AND THEIR IMPLEMENTATION   - Clear and consistent guidelines with adequate dissemination - Dissemination of guidelines by healthcare professional associations who facilitated workshops with health providers opinion leaders ("champions") - Adequate training and staffing policies and allocation –   *Hands-on training:* Observation of clinical practice and discussion platforms with birth simulation sessions, case reviews and team-building sessions |
| Shobo et al 2020 | HEALTH FACILITIES INFASTRUCTURE AND SUPPLIES   - Lack of privacy or quiet place to breastfeed – open rooming-in rooms that does not allow for privacy. Mothers dress up and do not breastfeed when relatives come to visit mothers after childbirth   HEALTH WORKER ENGAGEMENT   - Staffing shortages and workload- staffing shortages delayed health worker breastfeeding support and cleaning the rooming-in area, which delayed transfer of mother and infant there - Poor respectful maternity care – pregnant women denied safe traditional birth practices such as praying or reading religious texts during the second and third stages of labour were five times more likely not to breastfeed   within the first hour (RR=4.5, 95% CI 1.2-17.1) | POLICIES AND THEIR IMPLEMENTATION   - Adequate training and staffing policies and allocation –   *Hands-on training:* On-the-job training sessions with visiting clinical mentors, peer-to-peer discussions on the job  CAREGIVER ENGAGEMENT   - Acceptability and knowledge –   *Previous knowledge from ANC:* most mothers who practiced early breastfeeding said what they learned from ANC influenced their decision to do so   - Received postpartum health worker counselling and/or support – Encouragement from birth attendants as well as showing mothers how to place and breastfeed their newborns |
| Swarts et al 2010 | CAREGIVER ENGAGEMENT   - Misconceptions, beliefs and cultural practices -   *Harm*: Community perception that it is wrong for HIV+ mother to breastfeed as it will infect the baby   - Fear of HIV transmission or stigma –   *Stigma:* Fear of disclosure with any infant feeding practices that diverge from the norm | POLICIES AND THEIR IMPLEMENTATION   - Adequate training and staffing policies and allocation –   *BHFI training:* Supported health worker knowledge and provisioning of counselling for HIV+ mothers on breastfeeding  CAREGIVER ENGAGEMENT   - Acceptability and knowledge –   *Positive attitudes and familiarity:* The mothers that were breastfeeding their infants felt very positive about breastfeeding and mentioned many benefits of breastfeeding when asked what their feelings were about breastfeeding  *Receptiveness to health worker counselling:* Most of the women reported that counselling from health professionals had an influence on their infant feeding decision. |
| Tawiah-Agyemang et al 2008 | POLICIES AND THEIR IMPLEMENTATION   - Poor leadership and management structures - Changes in priority regions had a detrimental effect on the continuity of breast-feeding policy implementation. - Lack of guidelines/policies or their limited implementation - Although almost all policy makers and implementers were aware of the national breastfeeding policy, none of the health centers or hospitals visited during the study had any written guidelines on breast-feeding policies. Materials such as posters and ﬂip charts are also passed from national to regional to district to health facility level. At each level, a request often needs to be made before materials are assigned and district-level implementers and health workers may then need to make a trip to either the regional or district ofﬁce to collect materials.   HEALTH WORKER ENGAGEMENT   - Staffing shortages and workload – Although many health workers identiﬁed early initiation as important, counselling was reported to be rare due to staff shortages - Gaps in knowledge, misconceptions and inconsistent messaging –  *Rest*: When the traditional birth attendant or the midwife took the baby away for bathing or to rest, breast-feeding initiation was delayed usually by a few hours. Sometimes also felt the mother needed to rest   CAREGIVER ENGAGEMENT   - Misconceptions, beliefs and cultural practices - *Colostrum:* belief about colostrum and that breast milk arrives on the third day after birth - Insufficient milk production - the little breast milk they felt they had, it would be unfair to give the baby an empty breast or one with not enough milk to satisfy the child | POLICIES AND THEIR IMPLEMENTATION   - Adequate training and staffing policies and allocation –   *Pre-service training:* Health workers reported that they had been provided with information about initiation during their nursing or midwifery training. Health workers’ knowledge of the importance of early initiation was high. |
| Tiruye et al 2018 | CAREGIVER ENGAGEMENT   - Health conditions of mother/infant –  *Breast complications:* Breast engorgement, cracked nipple, inverted nipple, mastitis | CAREGIVER ENGAGEMENT   - Acceptability and knowledge –   *Positive attitudes and familiarity:* those who had previous breastfeeding experience (AOR 3.3; 95%CI 1.1, 10.7) had better breastfeeding technique practice   - Received postpartum health worker counselling and/or support - The practice of effective breastfeeding technique was significantly associated with mothers who had immediate breastfeeding technique counseling after birth (AOR 1.7, 95% CI 1.1, 2.8) and received information about attachment and positioning (AOR 1.8; 95% CI 1.1, 3). - Maternal characteristics –   *Education:* mothers who have educational status of secondary school and above (AOR 2.3; 95% CI 1.1, 3.9)   - Absence of breast problems - associated with higher rate of breastfeeding (AOR 4.0; 95% CI 1.4, 10.9) |
| Tongun et al 2018 | CAREGIVER ENGAGEMENT   - Misconceptions, beliefs and cultural practices - *Colostrum:* Discarding of colostrum associated with delay of initiation (aOR 9.89, 95% CI: 4.14-23.62)   *Formula:* Exposure to infant formula advertisement (aOR 1.82, 95% CI: 1.09 -3.02),   - Health conditions of mother/infant –  *Caesarean section*: Birth by caesarean section was associated with increased risk of delayed initiation (aOR 41, 95% CI: 12.21- 138) - Maternal characteristics –   *Martial status:* unmarried mothers associated with delay in initiation of breastfeeding (aOR 3.76, 95% CI: 1.53-9.24)  *House ownership:* no house ownership associated with delay in initiation of breastfeeding (aOR 1.52, 95% CI: 1.11-2.09) |  |
| van Rensburg et al 2016 | HEALTH WORKER ENGAGEMENT   - Gaps in knowledge, misconceptions and inconsistent messaging – *HIV+ mothers:* only a few (14.1%) of the healthcare workers included considered themselves to be experts regarding infant feeding and HIV *Overall gaps in knowledge:* only a few only (6.7%) could comprehensively explain the concept of exclusive breastfeeding support as described by the WHO - Gaps in practical skills and management of complications - a lack of practical application of knowledge among the healthcare workers as only about a third of health workers (36%) expressed very high level of confidence to be able to demonstrate easily to a mother how to breastfeed and less than a third (28.1%) on how to express the breast milk correctly | HEALTH WORKER ENGAGEMENT   - Good knowledge about breastfeeding benefits and practices –   *Infant feeding and HIV+:* Most respondents correctly indicated that infants who are formula fed have a higher risk for morbidity and mortality from causes other than HIV and infants who are breastfed usually have a much lower risk of dying from diarrhoea, acute respiratory infections and other diseases. Not even a single healthcare worker indicated that these mothers should not breastfeed due to the risk of HIV transmission.   - Respectful maternity care - Healthcare workers should be trained in counselling skills emphasizing the building of trust and encourage honesty in the healthcare worker/patient relationship. |
| West et al 2019 | HEALTH WORKER ENGAGEMENT   - Gaps in knowledge, misconceptions and inconsistent messaging – *HIV+ mothers:* Inconsistent messaging related to infant feeding for HIV positive mothers delivered by healthcare providers “I think breastfeeding is like falling pregnant, there are health care workers who would encourage an HIV positive woman to fall pregnant and there are those who won’t encourage HIV mothers to breastfeed because they are afraid that they will infect their babies.”–Nurse   CAREGIVER ENGAGEMENT   - Fear of HIV transmission or stigma –   *HIV transmission:* Even with counselling, for women who chose to formula feed, any element of HIV transmission risk outweighed the benefits of breastfeeding. Most women expressed a fear of transmission of HIV to their infants ““I didn’t want any chance for them [the infant] to get HIV. I felt that they [healthcare providers] said if you are positive and take your medication properly, then you can have a negative baby. I decided that I don’t want any chance.” |  |
| Yotebieng et al 2015 | CAREGIVER ENGAGEMENT   - Misconceptions, beliefs and cultural practices - *Giving water:* Giving water was identified as main contributor to suboptimum breastfeeding practices. However, distribution of flyers may have led to misunderstandings or incorrect advice.   *Cultural practices:* social customs identified in pretrial survey as main contributor to suboptimum practices. However, distribution of flyers may have led to misunderstandings or incorrect advice.   - Peer pressure by relatives and lack of mother decision-making power - engagement of family members through distribution of flyers may have led to misunderstandings or incorrect advice that were not suﬃciently countered by group counselling from nurses | POLICIES AND THEIR IMPLEMENTATION   - Adequate training and staffing policies and allocation –   *BHFI training:* 2 days’ intensive (16 h) didactic training using the WHO/UNICEF BFHI course   - Mechanisms of regulation and supportive supervision - During the month that followed the training, study personnel visited each clinic at least once a week to observe the trainees practise in real life conditions. At the end of each visit, a group and individual debrieﬁng was held to provide both collective and individual feedback. |
