# Supplementary material for: Barriers and facilitators for early and exclusive breastfeeding in health facilities in Sub-Saharan Africa: a systematic review
Source: Glob Health Res Policy. 2021 Jul 6;6:21. doi: 10.1186/s41256-021-00206-2 (PMC8259208; doi:10.1186/s41256-021-00206-2)
Supplement: Supplementary file 7 — Additional file 7: Table S7. Summary of themes by studies. [file 41256_2021_206_MOESM7_ESM.docx]

**Table S7: Summary of themes by studies**

|  | Barriers | Facilitators |
| --- | --- | --- |
| Health facilities infrastructure and supplies | **Overcrowding and lack of space** (Agbozo et al 2019, Amadhila and Rensburg 2020, Amsalu et al 2019, Kavle et al 2019, Moussa et al 2010, Nyati-Jokomo et al 2019)  **Lack of privacy or quiet place to breastfeed** (Chabeda et al 2020, Moussa et al 2010, Nyati-Jokomo et al 2019, Shobo et al 2020)  **Insufficient equipment or supplies** (Moussa et al 2010) | **Supplies that support breastfeeding practice** (Hasselberg et al 2016) |
| Supportive policies and policy implementation | **Poor leadership and management structures** (Agbozo et al 2019, Daniels and Jackson 2011, Nii Okai Aryeetey and Antwi 2013, Tawiah-Agyemang et al 2008)  **Inability to sustain skilled staff with due to staffing and training policies** (Agbozo et al 2019, Amadhila and Rensburg 2020, Amsalu et al 2019, Daniels and Jackson 2011, Dubik et al 2021, Ferguson et al 2009, Hasselberg et al 2016, Kavle et al 2019, Morhason-Bello et al 2009, Moussa et al 2010, Mphasha and Skaal 2019, Nabwera et al 2017, Nii Okai Aryeetey and Antwi 2013, Nikodem et al 1995)  **Lack of guidelines/policies or their limited implementation** (Chale et al 2016, Dubik et al 2021, Kavle et al 2019, Morgan and Jeggels 2015, Moussa et al 2010, Mphasha and Skaal 2019, Nabwera et al 2017, Nii Okai Aryeetey and Antwi 2013, Nikodem et al 1995, Okolo and Ogbonna 2002, Senbanjo et al 2014, Tawiah-Agyemang et al 2008) | **Commitment and leadership** (Agbozo et al 2019, Daniels and Jackson 2011, Kalisa et al 2015, Kavle et al 2019)  **Clear and consistent guidelines with adequate dissemination** (Agbozo et al 2019, Spira et al 2017)  **Mechanisms of regulation and supportive supervision** (Agbozo et al 2019, Ferguson et al 2009, Kavle et al 2019, Yotebieng et al 2015)  **Policy implementation: *rooming-in*** (Agbozo et al 2019, Fadupin et al 2020, Nii Okai Aryeetey and Antwi 2013), ***skin-to-skin*** (Moussa et al 2010), ***formula and/or mixed feeding*** (Moussa et al 2010, Morgan and Jeggels 2015)  **Adequate training and staffing policies and allocation: *increasing number of skilled staff*** (Awi and Alikor 2006, Ferguson et al 2009), ***hands-on training*** (Chale et al 2016, Dubik et al 2021, Kavle et al 2019, Shobo et al 2020, Spira et al 2017), ***BHFI training*** (Amadhila and Rensburg 2020, Daniels and Jackson 2011, Owoaje et al 2002, Swarts et al 2010, Yotebieng et al 2015), ***pre-service training*** (Dubik et al 2021, Tawiah-Agyemang et al 2008), ***task-shifting*** (Chabeda et al 2020, Moussa et al 2010, Kavle et al 2019) |
| Health worker engagement | **Staffing shortages and workload** (Amadhila and Rensburg 2020, Chabeda et al 2020, Daniels and Jackson 2011, Dubik et al 2021, Ferguson et al 2009, Kavle et al 2019, Morhason-Bello et al 2009, Moussa et al 2010, Nabwera et al 2017, Nii Okai Aryeetey and Antwi 2013, Shobo et al 2020, Tawiah-Agyemang et al 2008)  **Gaps in knowledge, misconceptions and inconsistent messaging*: formula*** (Agbozo et al 2019 , Nii Okai Aryeetey and Antwi 2013), ***pre-lacteal feeds*** ( Akuse and Obinya 2002, Moussa et al 2010, Okolo and Ogbonna 2002), ***infant feeding options for HIV+ mothers***  (Agbozo et al 2019, Amadhila and Rensburg 2020, Chaponda et al 2017, Lang'at et al 2018, Morgan and Jeggels 2015, Mphasha and Skaal 2019, Nabwera et al 2017, Nyawade et al 2016, van Rensburg et al 2016, West et al 2019), ***caesarean section mothers*** (Awi and Alikor 2006), ***rest*** (Tawiah-Agyemang et al 2008), ***potential harms*** (Mgolozeli et al 2019), ***overall gaps in knowledge*** (Chale et al 2016, Mphasha and Skaal 2019, Nikodem et al 1995, van Rensburg et al 2016)  **Gaps in practical skills and management of complications** (Daniels and Jackson 2011, Dubik et al 2021, Nii Okai Aryeetey and Antwi 2013, Okolo and Ogbonna 2002, van Rensburg et al 2016)  **Poor health worker attitude or willingness** (Daniels and Jackson 2011, Doherty et al 2019, Lang'at et al 2018, Mgolozeli et al 2019, Moussa et al 2010, Mphasha and Skaal 2019, Nii Okai Aryeetey and Antwi 2013, Nikodem et al 1995, Senbanjo et al 2014)  **Poor respectful maternity care** (Doherty et al 2019, Nikodem et al 1995, Chaponda et al 2017, Shobo et al 2020) | **Positive attitude and willingness for breastfeeding support** (Akuse and Obinya 2002, Dubik et al 2021, Ferguson et al 2009, Mgolozeli et al 2019, Mohamed et al 2018, Nikodem et al 1995, Nyawade et al 2016, Owoaje et al 2002)  **Good knowledge about breastfeeding benefits and practices: *infant feeding and HIV+*** (Mphasha and Skaal 2019, Owoaje et al 2002, van Rensburg et al 2016), ***management of complications and/or specialized care*** (Owoaje et al 2002), ***general*** (Mohamed et al 2018, Nikodem et al 1995, Owoaje et al 2002)  **Providing demonstrations and follow-up on breastfeeding practice** (Doherty et al 2019, Hasselberg et al 2016, Nikodem et al 1995, Nyawade et al 2016, Owoaje et al 2002)  **Providing respectful maternal care** (Hasselberg et al 2016, Nyawade et al 2016, van Rensburg et al 2016)  **Positive work culture and social norms among medical staff supporting breastfeeding** (Nyawade et al 2016) |
| Caregiver engagement | **Gaps in knowledge: *lack of or inadequate postpartum counselling*** (Amsalu et al 2019, Ighogboja et al 1996, Morgan and Jeggels 2015), ***lack of or inadequate counselling during ANC*** (Ighogboja et al 1996, Kalisa et al 2015, Kavle et al 2019, Mukerem and Haidar 2012),  **Misconceptions, beliefs and cultural practices: *giving water*** (Aghaji 2002, Kavle et al 2019, Moussa et al 2010, Yotebieng et al 2015), ***formula***  (Chaponda et al 2017, Tongun et al 2018), ***colostrum*** (Ighogboja et al 1996, Kalisa et al 2015, Tawiah-Agyemang et al 2008, Tongun et al 2018), ***needs rest*** (Kalisa et al 2015), ***harms*** (Kafulafula et al 2014, Nyati-Jokomo et al 2019, Swarts et al 2010), ***insufficient nutrition*** (Kusi-Amponsah Diji et al 2017), ***cultural traditions*** *(*Kavle et al 2019, Kusi-Amponsah Diji et al 2017, Lang'at et al 2018, Yotebieng et al 2015), ***negative attitudes in general*** (Mukerem and Haidar 2012)  **Insufficient milk production** (Aghaji 2002, Akuse and Obinya 2002, Chaponda et al 2017, Doherty et al 2019, Getnet et al 2020, Ighogboja et al 1996, Kalisa et al 2015, Kassa et al 2021, Kavle et al 2019, Kusi-Amponsah Diji et al 2017, Mukashyaka et al 2020, Olorunfemi and Dudley 2018, Tawiah-Agyemang et al 2008)  **Health conditions of mother/infant: *caesarean section*** (Awi and Alikor 2006, Getnet et al 2020, Hasselberg et al 2016, Mukashyaka et al 2020, Ighogboja et al 1996, Kalisa et al 2015, Kavle et al 2019, Tongun et al 2018), ***preterm or low birth weight*** (Degefa et al 2019, Hasselberg et al 2016), ***breast complications*** (Doherty et al 2019, Gejo et al 2019, Hasselberg et al 2016, Kusi-Amponsah Diji et al 2017, Mukerem and Haidar 2012, Olorunfemi and Dudley 2018, Tiruye et al 2018), ***illness of mother or infant*** (Fadupin et al 2020, Gejo et al 2019, Mukerem and Haidar 2012, Olorunfemi and Dudley 2018), ***maternal emotional stress*** (Kahindi et al 2020, Kusi-Amponsah Diji et al 2017)  **Difficulty with breastfeeding practice and receiving inadequate health worker support** (Kalisa et al 2015, Kavle et al 2019, Kusi-Amponsah Diji et al 2017, Moussa et al 2010, Mukashyaka et al 2020, Nyawade et al 2016)  **Peer pressure by relatives and lack of mother decision-making power** (Aghaji 2002, Chaponda et al 2017, Doherty et al 2019, Kalisa et al 2015, Lang'at et al 2018, Mukerem and Haidar 2012, Nabwera et al 2017, Nyati-Jokomo et al 2019, Olorunfemi and Dudley 2018, Yotebieng et al 2015)  **Fear of HIV transmission or stigma: *HIV transmission*** (Amadhila and Rensburg 2020, Gejo et al 2019, Iliyasu et al 2019, Kafulafula et al 2014, Kalisa et al 2015, Mukerem and Haidar 2012, Nyati-Jokomo et al 2019, Olorunfemi and Dudley 2018, Remmert et al 2020, West et al 2019), ***stigma*** (Lang'at et al 2018, Nabwera et al 2017, Nyati-Jokomo et al 2019, Swarts et al 2010)  **Maternal characteristics: *parity* (**Degefa et al 2019), ***education*** (Degefa et al 2019), ***age*** (Senghore et al 2018), ***marital status*** (Tongun et al 2018), ***house ownership***  *(*Tongun et al 2018) | **Acceptability and knowledge: *Positive attitudes and familiarity*** (Amsalu et al 2019, Getnet et al 2020, Kafulafula et al 2014, Kassa et al 2021, Olorunfemi and Dudley 2018, Senghore et al 2018, Swarts et al 2010, Tiruye et al 2018), ***previous knowledge from antenatal care*** (Degefa et al 2019, Doherty et al 2019, Getnet et al 2020, Ighogboja et al 1996, Kassa et al 2021, Nabwera et al 2017, Olorunfemi and Dudley 2018 Shobo et al 2020), ***receptiveness to health worker counselling*** (Morgan and Jeggels 2015, Mukerem and Haidar 2012, Swarts et al 2010)  **Received postpartum health worker counselling and/or support** (Awi and Alikor 2006, Doherty et al 2019, Fadupin et al 2020, Getnet et al 2020, Kahindi et al 2020, Kassa et al 2021, Ighogboja et al 1996, Iliyasu et al 2019, Morhason-Bello et al 2009, Nikodem et al 1995, Shobo et al 2020, Tiruye et al 2018)  **Learning skills and techniques to improve breastfeeding practice:** ***expressed breastmilk*** (Hasselberg et al 2016, Kahindi et al 2020, Nikodem et al 1995), ***timed strategies*** (Hasselberg et al 2016), ***to enhance nutrition*** (Hasselberg et al 2016)  **Supportive social networks and peer support groups:** ***HIV+ peers*** (Chaponda et al 2017, Mukerem and Haidar 2012, Nabwera et al 2017, Remmert et al 2020), ***peer*** (Hasselberg et al 2016), ***family*** (Hasselberg et al 2016, Morhason-Bello et al 2009, Mukerem and Haidar 2012, Olorunfemi and Dudley 2018, Remmert et al 2020, Senghore et al 2018)  **Maternal characteristics:** ***parity*** (Aghaji 2002), ***education*** (Aghaji 2002, Ojofeitimi et al 2000, Tiruye et al 2018), ***religion*** (Senbanjo et al 2014), ***private hospital attendance and/or socio-economic status*** (Senbanjo et al 2014, Senghore et al 2018)  **Absence of breast problems** (Tiruye et al 2018) |
